# *SPINK1*, *PRSS1*, *CTRC*, and *CFTR* Genotypes Influence Disease Onset and Clinical Outcomes in Chronic Pancreatitis

**DOI:** 10.1038/s41424-018-0069-5

**Published:** 2018-11-12

**Authors:** Wen-Bin Zou, Xin-Ying Tang, Dai-Zhan Zhou, Yang-Yang Qian, Liang-Hao Hu, Fei-Fei Yu, Dong Yu, Hao Wu, Shun-Jiang Deng, Jin-Huan Lin, An-Jing Zhao, Zhen-Hua Zhao, Hong-Yu Wu, Jia-Hui Zhu, Wei Qian, Lei Wang, Lei Xin, Min-Jun Wang, Li-Juan Wang, Xue Fang, Lin He, Emmanuelle Masson, David N. Cooper, Claude Férec, Zhao-Shen Li, Jian-Min Chen, Zhuan Liao

**Affiliations:** 1 Department of Gastroenterology, Changhai Hospital, Second Military Medical University, Shanghai, China; 2 Shanghai Institute of Pancreatic Diseases, Shanghai, China; 3 Key Laboratory of Arrhythmias of the Ministry of Education of China, East Hospital, Tongji University School of Medicine, Institute of Medical Genetics, Tongji University, Shanghai, China; 4 Medical Service Research Division, the Naval Medical Research Institute, Second Military Medical University, Shanghai, China; 5 Center for Translational Medicine, Second Military Medical University, Shanghai, China; 6 Department of Cell Biology, Center for Stem Cell and Medicine, Second Military Medical University, Shanghai, 200433 China; 7 Key Laboratory of Developmental Genetics and Neuropsychiatric Diseases (Ministry of Education), Bio-X Institutes, Shanghai Jiao Tong University, Shanghai, China; 8 UMR1078 ″Génétique, Génomique Fonctionnelle et Biotechnologies″, INSERM, EFS - Bretagne, Université de Brest, CHRU Brest, Brest, France; 9 Institute of Medical Genetics, School of Medicine, Cardiff University, Cardiff, UK

## Abstract

**Objectives:**

Rare pathogenic variants in the *SPINK1*, *PRSS1*, *CTRC*, and *CFTR* genes have been strongly associated with a risk of developing chronic pancreatitis (CP). However, their potential impact on the age of disease onset and clinical outcomes, as well as their potential interactions with environmental risk factors, remain unclear. These issues are addressed here in a large Chinese CP cohort.

**Methods:**

We performed targeted next-generation sequencing of the four CP-associated genes in 1061 Han Chinese CP patients and 1196 controls. To evaluate gene–environment interactions, the patients were divided into three subgroups, idiopathic CP (ICP; *n* = 715), alcoholic CP (ACP; *n* = 206), and smoking-associated CP (SCP; *n* = 140). The potential impact of rare pathogenic variants on the age of onset of CP and clinical outcomes was evaluated using the Kaplan–Meier model.

**Results:**

We identified rare pathogenic genotypes involving the *SPINK1*, *PRSS1*, *CTRC*, and/or *CFTR* genes in 535 (50.42%) CP patients but in only 71 (5.94%) controls (odds ratio = 16.12; *P* < 0.001). Mutation-positive patients had significantly earlier median ages at disease onset and at diagnosis of pancreatic stones, diabetes mellitus and steatorrhea than mutation-negative ICP patients. Pathogenic genotypes were present in 57.1, 39.8, and 32.1% of the ICP, ACP, and SCP patients, respectively, and influenced age at disease onset and clinical outcomes in all subgroups.

**Conclusions:**

We provide evidence that rare pathogenic variants in the *SPINK1*, *PRSS1*, *CTRC*, and *CFTR* genes significantly influence the age of onset and clinical outcomes of CP. Extensive gene–environment interactions were also identified.

## Introduction

Chronic pancreatitis (CP) is a chronic inflammatory process of the pancreas that leads to irreversible morphological changes and progressive impairment of both exocrine and endocrine functions^1^. Its prevalence is generally thought to be 30–50 per 100,000 individuals^1^ but may be as high as 120–143 per 100,000 individuals^2^. The disease is associated with a poor quality of life^3^, confers an increased risk of pancreatic cancer^4^, and represents a major cause of morbidity^5^. The leading cause of CP is excessive alcohol consumption (40–70% of cases^1^), followed by so-called idiopathic chronic pancreatitis (ICP; defined throughout this paper as the absence of any identifiable etiology prior to genetic analysis) that accounts for up to 25% of patients^6,7^.

It is generally thought that once CP is established, its progression cannot be reversed^1^. Thus, the identification of underlying heritable risk factors with strong effect holds out the promise of improved options in terms of prevention and treatment. Over the past two decades, it has been increasingly appreciated that ICP has a strong genetic basis, to which rare pathogenic variants (defined here as having a minor allele frequency of <1%^8,9^) in the *CFTR* (encoding cystic fibrosis transmembrane conductance regulator; MIM# 602421) gene^10,11^ and the three trypsin-dependent pathway genes^12^, namely *PRSS1* (encoding cationic trypsinogen; MIM# 276000)^13^, *SPINK1* (encoding pancreatic secretory trypsin inhibitor; MIM# 167790)^14^, and *CTRC* (encoding chymotrypsin C, MIM# 601405)^15,16^, make an important contribution^7,17,18^. Some rare pathogenic variants in these four genes have also been found to be overrepresented in patients with alcoholic chronic pancreatitis (ACP)^15,19–21^.

The above notwithstanding, most previous studies had a variety of limitations, including low numbers of patients screened, the analysis of only one or two genes, lack of appropriate control data from the normal population, the selective genotyping of known pathogenic variants or the sequencing of only a few specified exons and the limited evaluation of “other etiologic factors, particularly smoking, whose causative role has been more firmly established”^7^. These limitations have not only prevented a more accurate estimation of the global contribution of rare pathogenic variants in the four genes to CP but have also hampered our understanding of the complex gene–gene and gene–environment interactions in the disease. Moreover, the potential impact of rare pathogenic variants on the age of onset and severity of CP remains unclear. This latter issue has previously been addressed in the context of autosomal dominantly inherited hereditary pancreatitis, a rare cause of CP that accounts for <1% of cases^1^, but the three representative studies generated inconsistent findings^22–24^. Relevant studies in the context of ICP have so far been few and have invariably involved relatively small numbers of ICP patients (e.g., 35 in Sandhu and colleagues^25^, 45 in Xiao and colleagues^26^, and 61 in Cho and colleagues^27^), precluding firm conclusions. In the present study, we report findings from a comprehensive analysis of rare pathogenic vagrants in the *SPINK1*, *PRSS1*, *CTRC*, and *CFTR* genes in a large cohort of well-phenotyped Han Chinese patients with CP.

## Methods

### Participants and disease definitions

1061 CP patients, having neither a reported family history of the disease nor any of the following causative factors, namely post-traumatic, hypercalcemic, hyperlipidemic, and autoimmune, after comprehensive clinical and laboratory evaluations, participated in this study. It should be noted that, as in a previous study^7^, pancreas divisum was not considered here as a causative factor. CP was diagnosed in accordance with the Asia-Pacific consensus report^28^.

The participating patients were from 29 provinces and municipalities on the Chinese mainland, all of Han origin, and referred to the Department of Gastroenterology at Changhai Hospital between 2010 and 2015. We designed a questionnaire about detailed past medical and personal history including alcohol intake and cigarette smoking for patients with CP. These data were collected by face-to-face conversation during their hospital stay. Patients were divided into three subgroups as follows: ACP was assigned in terms of an alcohol intake of ≥80 g/d for a male and 60 g/d for a female for at least two years in accordance with previous publications^29,30^, irrespective of smoking status; among the remaining patients, those had smoked ≥20 cigarettes per day for at least two years were assigned to a “smoking-associated chronic pancreatitis (SCP)” subgroup; all the remaining patients were assigned to the “ICP” subgroup. A total of 1196 unrelated Han Chinese blood donors were used as healthy controls.

The electronic medical records of each patient were systematically evaluated for the following clinical features: age at disease onset (this was defined as the age at onset of abdominal pain; in patients who did not experience prior symptoms, age at diagnosis of CP); age at diagnosis of pancreatic stones, diabetes mellitus and steatorrhea; and M-ANNHEIM clinical stages. Pancreatic stones were diagnosed when stones or calcification were found in the pancreas by radiological examinations such as abdominal X-ray, CT, or MRI. Diabetes was diagnosed according to the diagnostic criteria of the American Diabetes Association, based on a threshold of ≥7.0 mmol/L for fasting plasma glucose or 2-h plasma glucose value of ≥11.1 mmol/L in a 75 g oral glucose tolerance test^31^. Steatorrhea was diagnosed in accordance with one of the following conditions: (1) chronic diarrhea with foul-smelling, oily bowel movements; (2) a positive result in a standard quantification test (a fecal fat excretion of over 14 g/d)^32^. M-ANNHEIM clinical stages were classified in accordance with ref. ^33^.

This study was approved by the Ethics Committee of Changhai Hospital. Written informed consent was obtained from all patients.

### Targeted next-generation sequencing of *PRSS1*, *SPINK1*, *CTRC*, and *CFTR*

We designed a total of 73 target-specific primer pairs (8 for *PRSS1*, 4 for *SPINK1*, 12 for *CTRC* and 49 for *CFTR*; see Supplementary Figures 1–4) for all exons and exon/intron boundaries of the four genes using Primer3^34^. The primers were synthesized with common adapter sequences at their 5′ ends as previously described^35^. The primer pairs were divided into two multiplex primer pools. Pre-amplification of the tagged-gene amplicons, generation of a barcoded DNA library for multiplex high-throughput sequencing, quantification and clean-up of the DNA library, and sequencing were performed essentially as previously described^35^. Variant calling and filtering for point mutations and micro-insertions or -deletions were performed as previously described^36^. All called rare variants were subjected to validation by Sanger sequencing (for details, see Supplementary Material). The nomenclature for the description of sequence variants followed HGVS recommendations^37^.

### Variant inclusion criteria

We focused on rare variants, defined as having a minor allele frequency of <1% in the control population^8,9^. Thus, we first excluded from consideration those variants with a minor allele frequency of ≥1% in the Chinese control dataset. Then, we divided the retained variants into known or novel categories by reference to data in the genetic risk factors in chronic pancreatitis database (http://www.pancreasgenetics.org/index.php) (for *SPINK1*, *PRSS1*, and *CTRC* variants) or *CFTR2* (https://www.cftr2.org/) and *CFTR*-France (https://cftr.iurc.montp.inserm.fr/cgi-bin/about_CFTR.cgi) databases (for *CFTR* variants). Of the known variants, those classified as pathogenic or disease-causing were included in the final analysis, whereas those classified as non-pathogenic were excluded from further consideration, and those classified as being of unknown significance were treated as novel variants. Of the novel variants, those affecting canonical splice sites or which resulted in premature stop codons or changes in the reading frame, were automatically classified as pathogenic and included in the final analysis. With regard to the novel missense variants detected, only those that were predicted to be pathogenic or likely pathogenic by at least five of six pathogenicity prediction algorithms, namely PolyPhen-2 (http://genetics.bwh.harvard.edu/pph2/index.shtml), PROVEAN (http://provean.jcvi.org/seq_submit.php), Mutationassessor (http://mutationassessor.org/r3/), SNPs&GO (http://snps.biofold.org/snps-and-go/snps-and-go.html), Align-GVGD (http://agvgd.hci.utah.edu/agvgd_input.php), and Mutationtaster (http://www.mutationtaster.org/) were included in the final analysis. All the other novel variants were excluded from consideration.

### Statistical analysis

The significance of the differences between variant or genotype carrier frequencies in patients and controls was tested by means of Chi-square or Fisher’s exact test using GraphPad Prism (v5.01). A *P* value < 0.05 was considered to provide evidence of statistical significance.

The following statistical analyses were performed using SPSS (v19.0) software. The statistical significance of differences between mutation-positive and -negative patients in terms of gender, pancreatic stones, diabetes mellitus, steatorrhea, and M-ANNHEIM clinical stages was assessed by means of the Chi-square or Fisher’s exact test; the odds ratio (OR) value with corresponding 95% confidence interval (CI) was calculated. The statistical significance of differences between mutation-positive and -negative patients in terms of the mean age at disease onset and the mean ages respectively at diagnosis of pancreatic stones, diabetes mellitus, and steatorrhea were compared using the *t* test under an equal condition by homogeneity test. The Kaplan–Meier method was used to plot survival curves. Differences between the survival curves of the tested groups were assessed using a log-rank test with a 0.05 significance level.

## Results

### Genetic analysis of *SPINK1*, *PRSS1*, *CTRC*, and *CFTR*

Targeted next-generation sequencing was performed on the entire coding sequences plus exon/intron boundaries of the five-exon *PRSS1*, four-exon *SPINK1*, eight-exon *CTRC*, and 27-exon *CFTR* genes in a total of 2257 Han Chinese subjects, including 1061 CP patients and 1196 controls. The age of subjects among the cases was 40.7 ± 16.0 years (range 6–85 years) and 47.7 ± 6.0 years (range 18–87 years) among the controls (*P* < 0.001). The CP group contained more males than the control group (69.9 vs 47.8%, *P* < 0.001). All called rare variants were subjected to Sanger sequencing and all validated variants were classified as being either pathogenic or non-pathogenic in accordance with the procedure described in the Methods. As such, we identified a total of 45 distinct rare pathogenic variants (11 in *SPINK1*, 6 in *PRSS1*, 12 in *CTRC,* and 16 in *CFTR*), of which 22 (5 in *SPINK1*, one in *PRSS1*, 7 in *CTRC,* and 9 in *CFTR*) had not been previously described (Supplementary Table 1). Below, we describe findings in the context of all patients, followed by findings in subgroup analyses and a genotype–phenotype relationship analysis in ICP.

### Confirmation of association with disease risk

We firstly evaluated the association of rare pathogenic variants with CP at both the single and the aggregate variant levels in the context of each gene, with OR values being provided both for those variants individually achieving a significant association and the aggregated variants. We robustly confirmed the individual associations of some previously reported rare pathogenic variants with CP (e.g., the most studied *SPINK1* c.101A>G (p.Asn34Ser), *PRSS1* c.365G>A (p.Arg122His), *CTRC* c.180C>T (p.Gly60Gly), and *CFTR* c.4056G>C (p.Q1352H)) and the association of the aggregated variants in each gene with CP. However, the variants differed markedly in terms of the strengths of their genetic effects. For example, the OR values of those variants individually achieving a significant association ranged from 3.24 (i.e., heterozygous *CFTR* c.4056G>C (p.Q1352H)) to 137 (i.e., homozygous *SPINK1* c.194+2T>C). The aggregated variants in the *CTRC*, *CFTR*, *PRSS1,* and *SPINK1* genes also differed quite dramatically in terms of the strength of their genetic effect, their corresponding ORs being 3.58, 3.71, 6.95, and 40.59, respectively (Supplementary Table 1).

### Impact on age of disease onset and clinical outcomes

To perform this task appropriately, we first stratified patients into genotype groups and generated OR values for those genotypes individually achieving a significant association with disease risk and the aggregated genotypes in each group (Supplementary Table 2). Altogether, rare pathogenic genotypes involving the *SPINK1*, *PRSS1*, *CTRC*, and/or *CFTR* genes were found in 535 (50.42%) of the 1061 CP patients but in only 71 (5.94%) of the 1196 controls (OR = 16.12; *P* < 0.001).

Next, we tested these pathogenic genotypes for possible association with disease onset and clinical outcomes in the patients. To this end, we compared the demographic and clinical characteristics of all CP patients with and without the pathogenic genotypes (Table 1). Mutation-positive patients had significantly earlier mean ages at disease onset, at diagnosis of pancreatic stones, and at diagnosis of diabetes mellitus. Mutation-positive patients were also more likely to have pancreatic stones (90.28 vs 78.14%) and to progress to M-ANNHEIM III/IV stages (7.66 vs 3.99%). Further, there were more females among the mutation-positive patients than among the mutation-negative patients. No significant differences were however observed between the two patient groups with respect to the relative number of patients having diabetes mellitus or steatorrhea (Table 1).

**Table 1:** Comparison of demographic and clinical characteristics in all Han Chinese CP patients with and without rare pathogenic genotypes involving *SPINK1*, *PRSS1*, *CTR*C, and/or *CFTR* genes

Characteristic	Pathogenic genotype-positive (*n* = 535)		Pathogenic genotype-negative (*n* = 526)		*P* value
	*n*	%	*n*	%	
Female	199	37.20	120	22.81	<0.001
Mean age at symptom onset (years ± SD)	29.70 ± 14.84		43.01 ± 15.97		<0.001
Pancreatic stones					
Yes	483	90.28	411	78.14	<0.001
Mean age at diagnosis (years ± SD)	34.22 ± 14.35		46.96 ± 14.18		<0.001
Diabetes mellitus					
Yes	122	22.80	129	24.52	0.51
Mean age at diagnosis (years ± SD)	39.33 ± 11.03		48.05 ± 11.22		<0.001
Steatorrhea					
Yes	93	17.38	73	13.88	0.12
Mean age at diagnosis (years ± SD)	38.69 ± 11.56		44.29 ± 13.03		0.004
M-ANNHEIM clinical stages					
I	361	67.48	345	65.59	0.51
II	133	24.86	160	30.42	0.04
III/IV	41	7.66	21	3.99	0.01

**Fig. 1:**
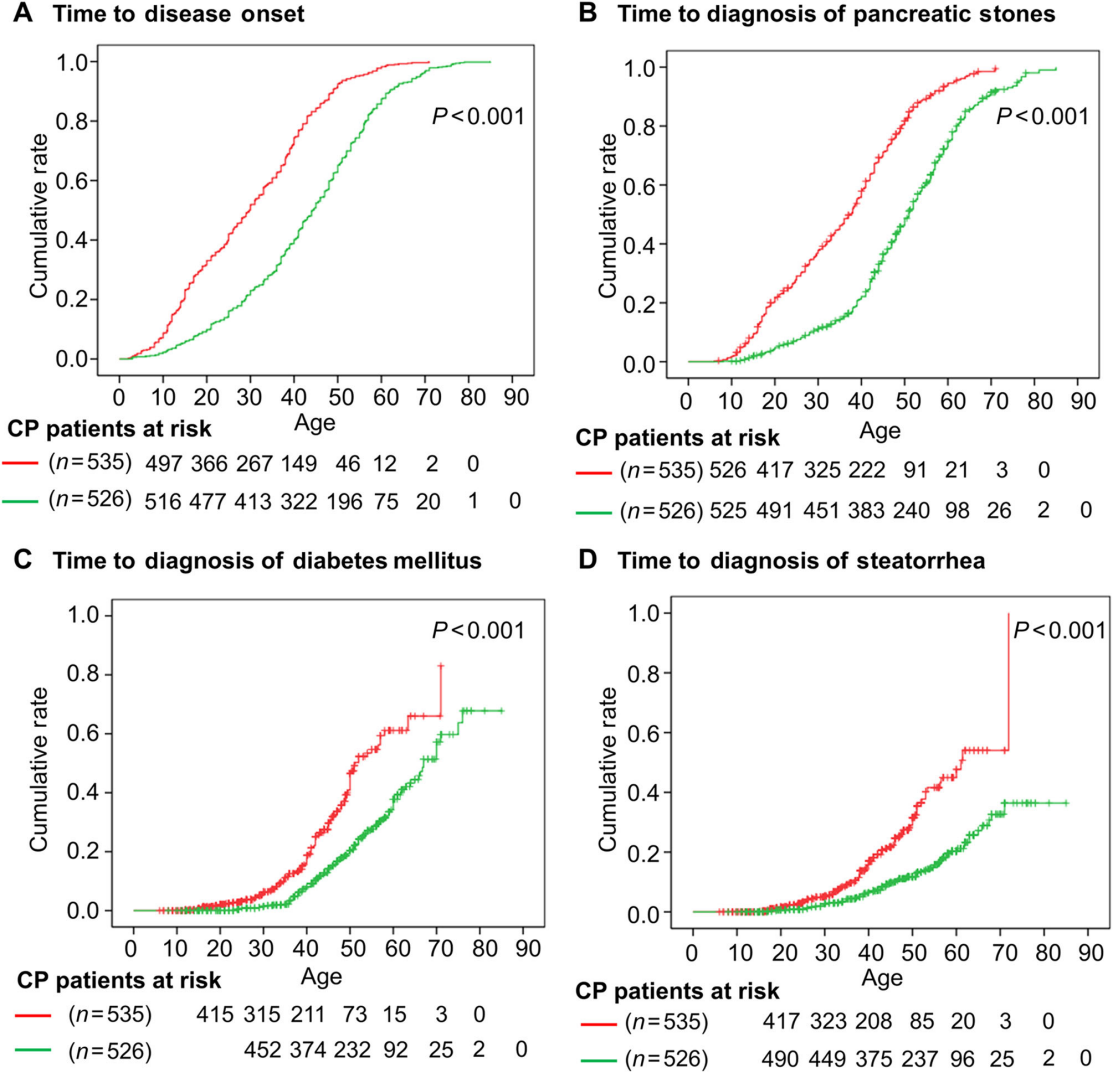
Pathogenic genotypes affect disease onset and clinical outcomes of CP. Kaplan–Meier plots of age at disease onset (**a**), age at diagnosis of pancreatic stones (**b**), age at diagnosis of diabetes mellitus (**c**), and age at diagnosis of steatorrhea (**d**) for all Han Chinese CP patients with and without pathogenic *SPINK1*, *PRSS1*, *CTRC*, and/or *CFTR* genotypes. Red, patients with pathogenic genotypes. Blue, patients without pathogenic genotypes

Moreover, using the Kaplan–Meier model, we demonstrated that the median ages at disease onset of CP, at diagnosis of pancreatic stones and at diagnosis of diabetes mellitus in the mutation-positive ICP patients were respectively 14.4 years [29.8 (95% CI, 27.7–31.9) vs 44.2 (95% CI, 42.4–46.0)], 13.6 years [37.4 (95% CI, 35.6–39.2) vs 51.0 (95% CI, 49.3–52.7)], and 15.9 years [51.1 (95% CI, 47.6–54.6) vs 67.0 (95% CI, 63.0–71.0)] earlier than in the mutation-negative ICP patients. Moreover, at 60 years of age, the cumulative incidence of steatorrhea was 57.7% for mutation-positive patients but only 31.0% for mutation-negative patients (Fig. 1).

### Comparative analyses across three subgroups

In an attempt to acquire a flavor of the gene–environment interactions in CP, we compared the overall prevalence of rare genotypes and their impact on disease onset and severity across three subgroups, ICP (*n* = 715; 318 females, 397 males), ACP (*n* = 206; one female, 205 males), and SCP (*n* = 140; all males). Pathogenic genotype distribution in ICP patients and controls, and OR values wherever appropriate, as performed for all patients, are provided in Table 2. As for the comparatively small ACP and SCP cohorts, only the distributions of pathogenic genotypes in patients are provided (Supplementary Table 3).

**Table 2:** Rare pathogenic genotypes involving the *SPINK1*, *PRSS1*, *CTRC*, and/or *CFTR* genes in Han Chinese ICP patients and controls

Gene(s)	Genotype^a^	ICP patients (*n* = 715)		Controls (*n* = 1196)		OR	95% CI	*P* value
		+	%	+	%			
*SPINK1* only	c.[93_101del];[=]	2	0.28	0	0			NS
	c.[101A>G];[=]	7	1.12	5	0.42			NS
	c.[142G>A];[=]	1	0.14	0	0			NS
	c.[174C>A];[=]	0	0	1	0.08			NS
	c.[194G>A];[=]	1	0.14	0	0			NS
	c.[194+2T>C];[=]	179	25.03	13	1.09	30.39	17.15–53.86	<0.001
	c.[202C>T];[=]	1	0.14	0	0			NS
	c.[101A>G];[101A>G]	1	0.14	0	0			NS
	c.[194+2T>C];[194+2T>C]	45	6.29	0	0	162.4	9.98–2642	<0.001
	c.194+2T>C(;)93_101del	1	0.14	0	0			NS
	c.194+2T>C(;)101A>G	9	1.26	0	0	32.18	1.87–554.1	<0.001
	c.194+2T>C(;)172T>A	1	0.14	0	0			NS
	c.194+2T>C(;)206C>T	3	0.42	0	0			NS
	Subtotal	251	37.76	19	1.59	33.51	20.76–54.08	<0.001
*PRSS1* only	c.[86A>T];[=]	7	0.98	0	0	25.33	1.44–444.5	0.002
	c.[346C>T];[=]	7	1.12	0	0	28.75	1.66–499.2	0.001
	c.[364C>T];[=]	3	0.42	0	0			NS
	c.[365G>A];[=]	16	2.24	3	0.25	9.69	2.83–33.18	<0.001
	c.[544A>T];[=]	1	0.14	0	0			NS
	c.[623G>C];[=]	19	3.36	22	1.84	1.85	1.03–3.33	0.036
	c.346C>T(;)623G>C	1	0.14	0	0			NS
	Subtotal	54	7.55	25	2.09	3.90	2.41–6.32	<0.001
*CTRC* only	c.[2T>C];[=]	0	0	1	0.08			NS
	c.[94G>A];[=]	1	0.14	0	0			NS
	c.[176G>A];[=]	0	0	1	0.08			NS
	c.[180C>T];[=]	4	0.56	3	0.25			NS
	c.[217G>T];[=]	0	0	1	0.08			NS
	c.[760C>T];[=]	0	0	2	0.17			NS
	c.649G>A(;)703G>A	1	0.14	0	0			NS
	Subtotal	6	0.84	8	0.67			NS
*CFTR* only	c.[263T>G];[=]	0	0	1	0.08			NS
	c.[1488G>T];[=]	2	0.28	0	0			NS
	c.[1549T>C];[=]	0	0	1	0.08			NS
	c.[1630G>T];[=]	1	0.14	0	0			NS
	c.[1813T>C];[=]	0	0	1	0.08			NS
	c.[1858C>T];[=]	0	0	1	0.08			NS
	c.[1865G>A];[=]	1	0.14	1	0.08			NS
	c.[2909G>A];[=]	4	0.56	1	0.08			NS
	c.[3205G>A];[=]	3	0.42	1	0.08			NS
	c.[3635delT];[=]	1	0.14	0	0			NS
	c.[3987_3988del];[=]	0	0	1	0.08			NS
	c.[4056G>C];[=]	8	1.12	11	0.92			NS
	Subtotal	20	2.80	19	1.59			NS
*SPINK1* and *PRSS1*	*SPINK1*:c.[194+2T>C] *PRSS1*:c.[86A>T]	1	0.14	0	0			NS
	*SPINK1*:c.[194+2T>C];[194+2T>C] *PRSS1*:c.[365G>A]	1	0.14	0	0			NS
	*SPINK1*:c.[194+2T>C] *PRSS1*:c.[623G>C];[623G>C]	1	0.14	0	0			NS
	*SPINK1*:c.[194+2T>C] *PRSS1*:c.[623G>C]	31	4.34	0	0	110.1	6.72–1804	<0.001
	Subtotal	34	4.76	0	0	121.1	7.41–1980	<0.001
*SPINK1* and *CTRC*	*SPINK1*:c.[194+2T>C] *CTRC*:c.[180C>T]	8	1.12	0	0	28.75	1.66–499.2	0.001
	*SPINK1*:c.[194+2T>C] *CTRC*:c.[181G>A]	1	0.14	0	0			NS
	*SPINK1*:c.[194+2T>C] *CTRC*:c.[493+1G>A]	1	0.14	0	0			NS
	*SPINK1*:c.[194+2T>C] *CTRC*:c.[641G>A]	1	0.14	0	0			NS
	Subtotal	11	1.54	0	0	39.06	2.30–664.4	
*PRSS1* and *CTRC*	*PRSS1*:c.[365G>A] *CTRC*:c.[94G>A]	1	0.14	0	0			NS
	*PRSS1*:c.[365G>A] *CTRC*:c.[180C>T]	1	0.09	0	0			NS
*SPINK1* and *CFTR*	*SPINK1*:c.[101A>G] *CFTR*:c.[2909G>A]	1	0.14	0	0			NS
	*SPINK1*:c.[194+2T>C] *CFTR*:c.[2909G>A]	2	0.28	0	0			NS
	*SPINK1*:c.[194+2T>C] *CFTR*:c.[2936A>C]	2	0.28	0	0			NS
	*SPINK1*:c.[194+2T>C] *CFTR*:c.[3205G>A]	2	0.28	0	0			NS
	*SPINK1*:c.[194+2T>C] *CFTR*:c.[4056G>C]	10	0.28	0	0			
	*SPINK1*:c.[194+2T>C];[194+2T>C] *CFTR*:c.[4056G>C]	1	0.14	0	0			NS
	*SPINK1*:c.194+2T>C](;)199C>T *CFTR*:c.[2173G>A]	1	0.14	0	0			NS
	Subtotal	19	0.27	0	0	67.0	4.04–1112	<0.001
*PRSS1* and *CFTR*	*PRSS1*:c.[346C>T] *CFTR*:c.[4056G>C]	1	0.14	0	0			NS
	*PRSS1*:c.[623G>C] *CFTR*:c.[2936A>C]	1	0.14	0	0			NS
	*PRSS1*:c.[623G>C] *CFTR*:c.[3205G>A]	3	0.42	0	0			NS
	*PRSS1*:c.[623G>C] *CFTR*:c.[4056G>C]	1	0.14	0	0			NS
	Subtotal	6	0.84	0	0	21.92	1.23–390.0	0.0027
*CTRC and CFTR*	*CTRC*:c.[180C>T] *CFTR*:c.[3205G>A]	2	0.28	0	0			NS
	*CTRC*:c.[180C>T] *CFTR*:c.[4056G>C]	1	0.14	0	0			NS
	Subtotal	3	0.42	0	0			NS
*SPINK1, PRSS1,* and *CFTR*	*SPINK1*:c.[194+2T>C] *PRSS1*:c.[623G>C] *CFTR*:c.[4056G>C]	2	0.28	0	0			NS
	Total	408	57.06	71	4.35	21.06	15.88–27.92	<0.001

*NS* not significant

^a^Nomenclature following HGVS recommendations (http://varnomen.hgvs.org/)^37^

**Fig. 2:**
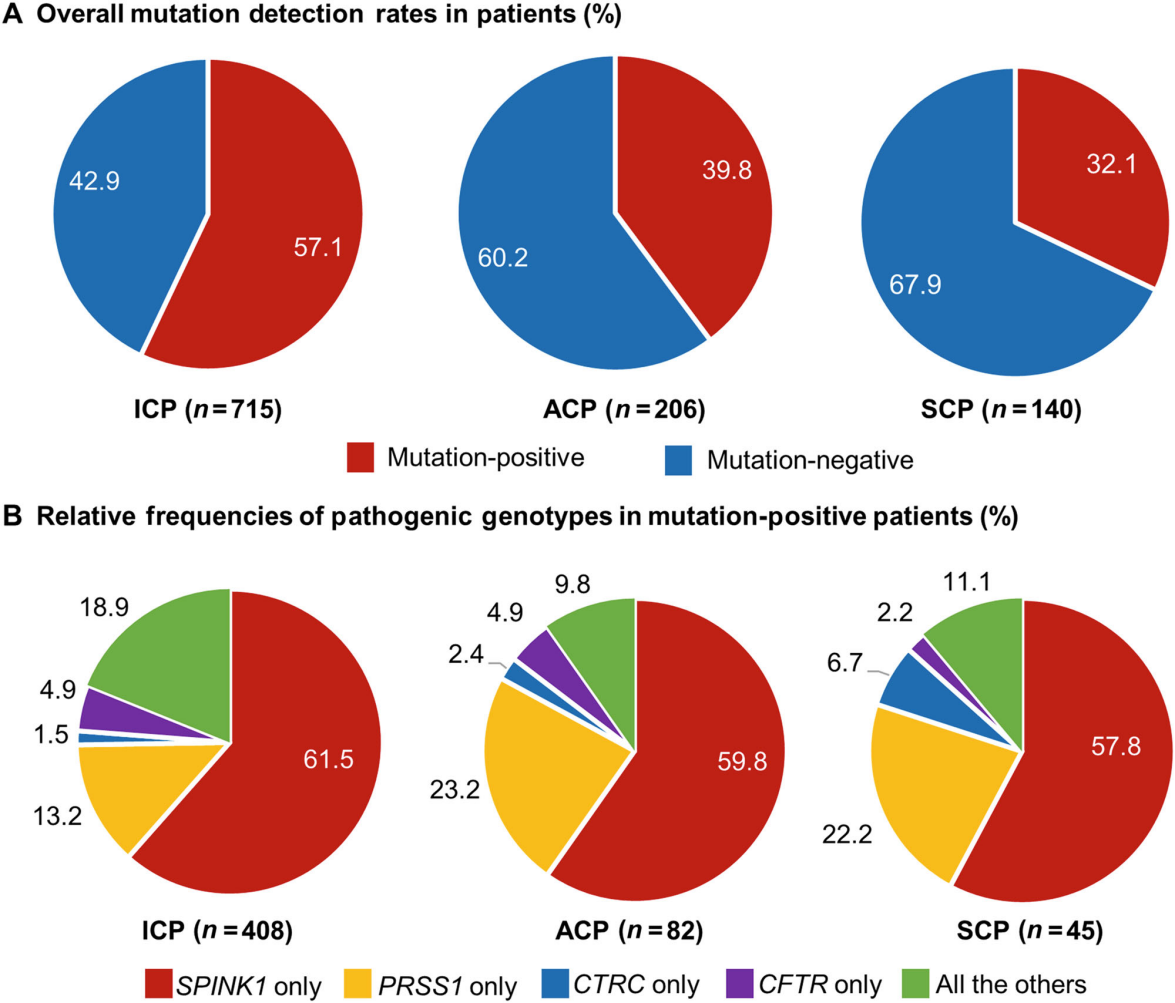
Comparison of overall prevalence and relative distribution of pathogenic genotypes in Han Chinese ICP, ACP and SCP patients. Data from all patients are also included for the purposes of comparison. The genotypes are defined as in Table 2 and Supplementary Table 3

Pathogenic genotypes were found in 57.1% (408/715) of ICP patients (Table 2), which is significantly higher than the 39.8% (82/206) detection rate in ACP patients (Supplementary Table 3; *P* < 0.0001), the 32.1% (45/140) detection rate in SCP patients (Supplementary Table 3; *P* < 0.0001) as well the 50.4% (535/1061) detection rate in all patients (Supplementary Table 2; *P* = 0.006) (see also Fig. 2a). In terms of the relative frequencies of different pathogenic genotypes among the three subgroups of mutation-positive patients, the most obvious observations are a ~10% decrease in the “*PRSS1* only” genotypes and an ~8% increase in the “all the others” genotypes in ICP than either ACP or SCP (Fig. 2b). Nonetheless, no significant differences were apparent among the three subgroups of mutation-positive patients in terms of the relative detection rates of the most frequent “*SPINK1* only” genotypes (i.e., 61.5% (251/408) in ICP, 59.8% (49/82) in ACP, and 57.8% (26/45) in SCP; Table 2 and Supplementary Table 3, Fig. 2b) and the most frequent single genotype, *SPINK1*:c.[194+2T>C];[=] (i.e., 43.9% (179/408) in ICP, 47.6% (39/82) in ACP and 46.7% (21/45) in SCP; Table 2 and Supplementary Table 3)

.

Using the Kaplan–Meier model, we demonstrated that pathogenic genotypes affected the age of disease onset and clinical outcomes across the three subgroups, with the strongest effects being observed in the ICP patients (Supplementary Figure 5; Supplementary Table 4).

### Genotype and phenotype relationships in the context of ICP

**Fig. 3:**
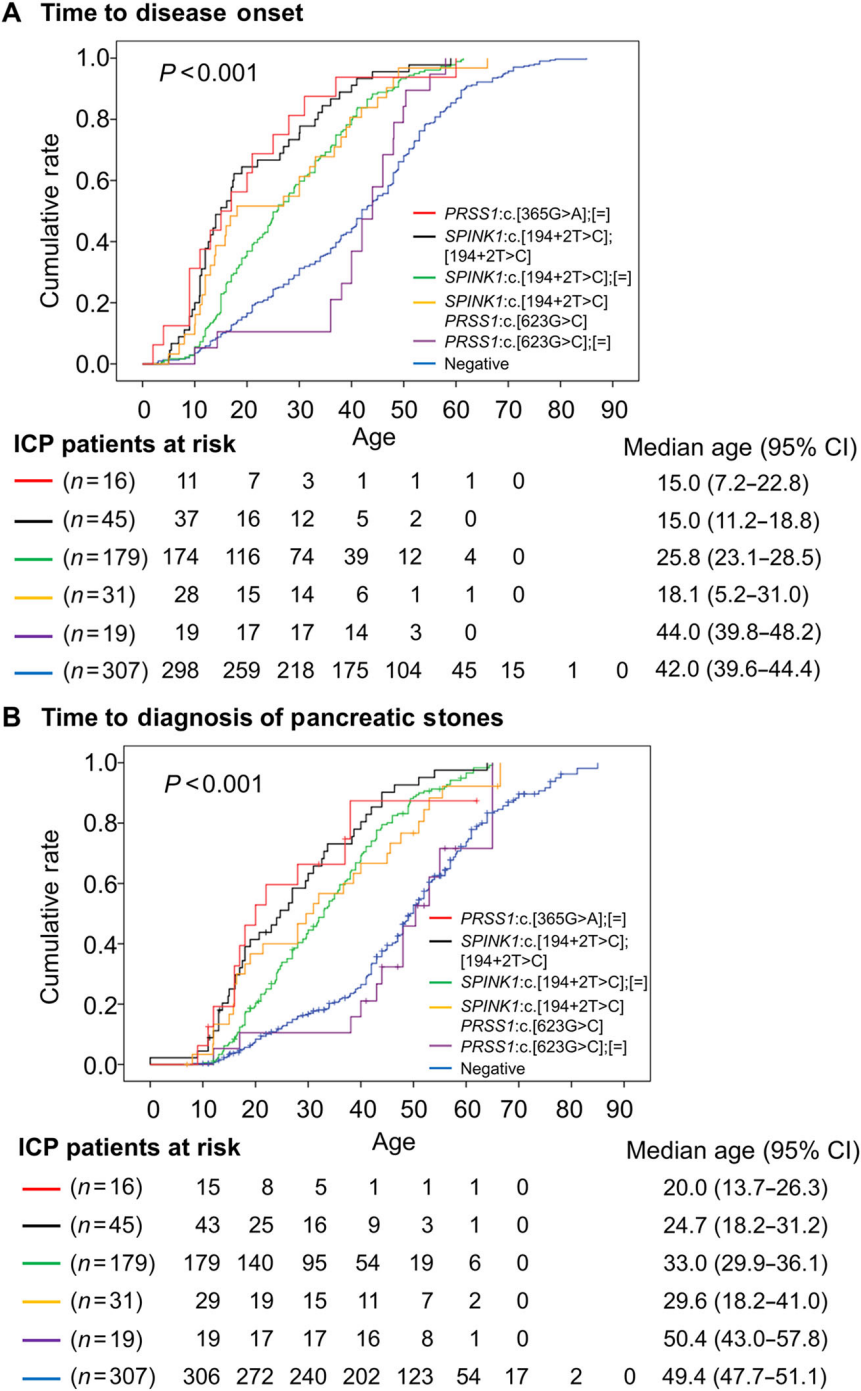
Impact of different pathogenic genotypes on disease onset and cumulative rate of pancreatic stones in ICP. Kaplan–Meier plots of age at disease onset (**a**) and of age at diagnosis of pancreatic stones (**b**) for the four most frequent pathogenic genotypes in the Han Chinese ICP patients. *PRSS1* c.365G>A (p.Arg122His) was included for the purposes of comparison

As shown in Table 2, the four most frequent genotypes were *SPINK1*:c.[194+2T>C];[=] (i.e., *SPINK1* c.194+2T>C heterozygote; *n* = 179), *SPINK1*:c.[194+2T>C];[194+2T>C] (i.e., *SPINK1* c.194+2T>C homozygote; *n* = 45), *SPINK1*:c.[194+2T>C] *PRSS1*:c.[623G>C] (i.e., *SPINK1* c.194+2T>C and *PRSS1* c.623G>C (p.Gly208Ala) *trans*-heterozygotes; *n* = 31), and *PRSS1*:c.[623G>C];[=] (i.e., *PRSS1* c.623G>C heterozygote; *n* = 19). Most notably, *SPINK1* c.194+2T>C in homozygosity exhibited a more than fivefold higher OR than when it was present in the heterozygous state (162.4 vs 30.39). Moreover, the *PRSS1* c.623G>C variant, when present in the heterozygous state, was associated with an OR of 1.85; however, its co-inheritance with *SPINK1* c.194+2T>C served to increase the OR associated with *SPINK1* c.194+2T>C heterozygosity from 30.39 to 110.1 (Table 2). These four most frequent genotypic combinations manifesting quite different ORs provided us with a unique opportunity to explore genotype–phenotype relationships in ICP. We therefore employed Kaplan–Meier analysis to compare the impact of the different genotypes with respect to median age at disease onset (Fig. 3a). We found no significant difference either between ICP patients harboring *PRSS1* c.[623G>C];[=] and mutation-negative ICP patients (*P* =0.26) or between ICP patients harboring the *SPINK1*:c.[194+2T>C] *PRSS1*:c.[623G>C] genotype and patients with the *SPINK1* c.[194+2T>C];[=] genotype (*p* = 0.78). However, the median age at disease onset in patients with either *SPINK1* c.[194+2T>C];[=] or *SPINK1*:c.[194+2T>C] *PRSS1*:c.[623G>C] was significantly earlier than that in mutation-negative patients (*P* < 0.001). Patients harboring *SPINK1*:c.[194+2T>C];[194+2T>C] exhibited the earliest median age at disease onset, 10 years earlier than that exhibited by ICP patients harboring *SPINK1*:c.[194+2T>C];[=] and 27 years earlier than that in mutation-negative patients (Fig. 3a). Similar differences were observed with the Kaplan–Meier analysis with respect to the age at diagnosis of pancreatic stones (Fig. 3b). Furthermore, *PRSS1*:c.[365G>A];[=] (i.e., *PRSS1* c.365G>A (p.Arg122His) heterozygote; *n* = 16) was the fifth most frequent genotype in the ICP cohort (Table 2). It displayed a very similar impact to that of *SPINK1*:c.[194+2T>C];[194+2T>C] in terms of both time to disease onset (*P* = 0.99) and time to diagnosis of pancreatic stones (*P* = 1.0) by means of Kaplan–Meier analysis (Fig. 3).

## Discussion

We have recently demonstrated marked ethnic differences in genetic predisposition to CP between Han Chinese and European populations in the context of three pancreatitis susceptibility loci, namely the absence of the *CEL-HYB* risk allele^38^ in the Chinese population^39^, the lack of significant enrichment of rare pathogenic *CPA1* variants^40^ in Chinese ICP patients^36^, and the lack of any contribution of the *CTRB1-CTRB2* risk allele^41^ to CP risk variation in the Chinese population due to allele near-fixation^42^. These risk variants were therefore not considered in the present study. In addition, we focused only on rare variants. Thus, common CP-predisposing polymorphisms^30,43^ were not considered.

The constellation of the concurrent and comprehensive sequence analysis of all exons and exon/intron boundaries of the *SPINK1*, *PRSS1*, *CTRC*, and *CFTR* genes, the employment of stringent criteria for classifying rare pathogenic variants, the use of large numbers of well-phenotyped CP patients with detailed demographic and clinical characteristics, and the use of a large number of healthy controls, allowed us not only to robustly replicate the disease association originally noted in European populations of rare pathogenic variants in each gene studied, but also to identify multiple novel variants in the Han Chinese population. More importantly, these factors allowed us to come up with several new findings, some of which are discussed here.

*SPINK1* p.N34S is the variant most frequently associated with ICP in European countries, being present in 382 of 5962 (6.4%) combined Caucasian ICP patient alleles and 83 of 11638 (0.07%) combined control alleles (OR = 9.70)^20^. It is pertinent to point out that *SPINK1* p.N34S itself has no functional effect but is in complete (in Caucasians) or high (in Han Chinese) linkage disequilibrium with a functional regulatory single nucleotide polymorphism in an upstream enhancer^44^. In the Han Chinese population, *SPINK1* p.N34S was present in only 19 of 1430 (1.33%) ICP patient alleles and 5/2392 (0.21%) control alleles (OR = 6.4; *P* < 0.001), and was much less common than the most frequent variant, *SPINK1* c.194+2T>C, whose allele frequency was 350/1430 (24.5%) in ICP patients and 13/2392 (0.54%) in controls (OR = 59.3; Table 2). Put another way, *SPINK1* c.194+2T>C-harboring genotypes (including simple heterozygotes, simple homozygotes, compound heterozygotes, and *trans*-heterozygotes) were found in up to 303 (42.4%) of the 715 Han Chinese ICP patients (Table 2). *SPINK1* c.194+2T>C affects gene function by disrupting the canonical donor splice site of intron 2^45,46^. Consistent with this profound functional effect, *SPINK1* c.194+2T>C heterozygotes were significantly associated with both disease onset and clinical outcomes, whereas homozygosity for *SPINK1* c.194+2T>C was associated with an even stronger effect than heterozygosity for *SPINK1* c.194+2T>C (Fig. 3). These findings, considered together with the extremely high OR values associated with *SPINK1* c.194+2T>C heterozygosity, established that *SPINK1* c.194+2T>C is the most important clinically actionable genetic risk factor for CP in the Han Chinese population. Additionally, it is pertinent to point out that *SPINK1*:c.[194+2T>C];[194+2T>C] actually had a similar impact on disease onset and clinical outcomes to *PRSS1*:c.[365G>A];[=] (Fig. 3), the most common genotype causing hereditary pancreatitis^13^. These observations serve to identify *SPINK1*:c.[194+2T>C];[194+2T>C] and *PRSS1*:c.[365G>A];[=] as the most severe genotypes and *SPINK1*:c.[194+2T>C];[=] to be a severe genotype predisposing to CP.

The simultaneous analysis of the *SPINK1*, *PRSS1*, *CTRC*, and *CFTR* genes had several advantages over the analysis of each gene independently. First, it allowed direct comparison of the relative contributions of rare pathogenic variants in each of the four genes to CP. In this regard, it is interesting to note that when the pathogenic variants were stratified into unique genotypes, neither the “*CTRC* only” genotypes nor the “*CFTR* only” genotypes (both at the individual genotype level and the aggregated genotype level) showed a significant association with CP (Table 2; Supplementary Table 2), a new indication of the generally less important contribution of *CTRC* and *CFTR* to CP as compared to *SPINK1* and *PRSS1*. Second, it allowed the ascertainment of gene–gene interactions in the disease; thus, for example, *trans*-heterozygotes (*n* = 77) accounted for 18.8% of the 408 rare pathogenic genotypes found in the 715 ICP patients (Table 2). Third, it allowed the identification of novel genotype–phenotype relationships, as exemplified by the findings illustrated in Fig. 3.

Excessive alcohol intake has long been established to be the primary cause of CP^1^. By contrast, only relatively recently has cigarette smoking been identified as an independent etiological risk factor for CP^1,47–51^. Nonetheless, there are currently no consensus guidelines as to when CP should be considered to have been caused by either alcohol intake or cigarette smoking. To deal with this issue, we adopted a pragmatic approach to divide our patients into three working subgroups, ICP, ACP, and SCP, an indispensable starting point for investigating gene and environment interactions in CP. Clearly, it will be necessary to refine these subgroup definitions as further data become available.

Our three subgroup cohorts are, respectively, by far the largest to date in which all exons and exon/intron boundaries of the *SPINK1, PRSS1*, *CTRC*, and *CFTR* genes have been simultaneously analyzed for rare pathogenic variants. This is also the first time that an “SCP” cohort has been subjected to extensive genetic analysis. The comparative analysis across the three subgroups revealed several important and novel findings. First, the ICP cohort differed significantly from both ACP and SCP cohorts in terms of the four survival curves (Supplementary Figure 5). For example, both mutation-positive ACP and SCP patients appeared to acquire the disease later and to have pancreatic stones, diabetes mellitus and steatorrhea later than mutation-positive ICP patients. These apparently paradoxical observations may be explicable by assuming that many children and adolescents, once they had experienced symptoms of pancreatitis or had been diagnosed with pancreatitis, would have avoided heavy alcohol consumption and/or cigarette smoking in later years and hence would rarely have been diagnosed with ACP or SCP as adults. An alternative and non-mutually exclusive explanation might be that the pathological mechanism(s) underlying ACP and SCP are qualitatively different from that of ICP^52,53^. Irrespective of the precise underlying reason(s), rare pathogenic genotypes were found to significantly affect the age of disease onset and the occurrence of multiple clinical outcomes in ACP and SCP, suggestive of a possible synergistic effect of genetic risk factors, alcohol consumption and cigarette smoking upon the pathogenesis of CP. Moreover, the impact of pathogenic genotypes on disease onset and clinical outcomes in ACP appeared to be less profound than that in SCP (Supplementary Figure 5). In this regard, it is pertinent to make two points. First, under our subgroup classification approach, ACP was first assigned irrespective of smoking status. Consistent with the phenomenon that alcoholics often also tend to be smokers, more than 80% of our ACP patients fitted the subgroup definition of SCP. This co-existence of two environmental risk factors in most of the ACP patients may have rendered the impact of genetic factors less visible. Second, we used a different criterion to classify SCP, namely those CP patients who had smoked ≥12 pack-years were designated as SCP. Using this new criterion, 159 patients were assigned to SCP and 49 of these were mutation-positive. Kaplan–Meier analysis of the reclassified SCP as well as ICP cohorts did not alter our main conclusions in any way. Irrespective of how SCP and ACP were classified, the fact that rare pathogenic genotypes in the four genes were found in 32–40% of the ACP and SCP subgroup patients (Supplementary Table 3), suggested an unprecedented degree of gene–environment interactions in CP.

The above notwithstanding, our study has certain limitations. For example, we did not analyze gross genomic rearrangements or copy number variants, a rare category of variant that can cause or predispose to CP^54^. Although we adopted a rather stringent approach to classifying newly identified variants as pathogenic or non-pathogenic (with newly detected missense variants for example, only those that were predicted to be pathogenic or likely pathogenic by at least five of six pathogenicity prediction algorithms were included for analysis), it is still possible that some of these putatively pathogenic variants were incorrectly annotated. However, we consider it unlikely that this affected the main conclusion because all the newly classified pathogenic variants were individually rare in our cohorts.

In conclusion, on the basis of comprehensive sequencing in a large number of well-phenotyped Han Chinese participants, we have provided evidence that rare pathogenic variants in the *SPINK1*, *PRSS1*, *CTRC*, and *CFTR* genes significantly affect the onset and clinical outcomes of CP across three subgroups. We have also identified extensive gene–environment interactions in the disease and uncovered novel genotype–phenotype relationships. The findings from this study should have important implications for genetic testing and counseling, personalized medicine and prognosis of CP.

## Study Highlights

### What is current knowledge

- Genetic variants in *SPINK1*, *PRSS1*, *CTRC*, and *CFTR* predispose to chronic pancreatitis.
- The potential impact of these genetic variants on age at disease onset and clinical outcomes remains unclear.
- The potential interactions of these genetic variants with environmental risk factors also remain unclear.

### What is new here

- Genetic variants in the four genes were found to influence age of disease onset and clinical outcomes in chronic pancreatitis.
- We demonstrated significant gene–environment interactions in chronic pancreatitis.

## Electronic supplementary material

Supplementary Information
