# Supplementary material for: SPINK1, PRSS1, CTRC, and CFTR Genotypes Influence Disease Onset and Clinical Outcomes in Chronic Pancreatitis
Source: Clin Transl Gastroenterol. 2018 Nov 12;9(11):204. doi: 10.1038/s41424-018-0069-5 (PMC6232107; doi:10.1038/s41424-018-0069-5)
Supplement: Supplementary file 1 — Supplementary Information [file 41424_2018_69_MOESM1_ESM.docx]

**Supplementary Material**

*Variant Validation by Sanger Sequencing*

All called rare variants were subjected to validation by Sanger sequencing. Sequences of the primers used to amplify the fragments of interest are provided in Supplementary Figures 1-4. PCR was performed in a 25-μl reaction mixture containing 12.5 µL HotStarTaq Master Mix (Qiagen, Germany), 0.4 µM of each of the corresponding forward and reverse primers, and 1 μl DNA (10-50 ng/μL). The PCR program comprised an initial denaturation at 95°C for 15 min, followed by 30 cycles of denaturation at 95°C for 30 s, annealing at 54°C-64°C for 30 s, and extension at 72°C for 2 min, and a final extension step at 72°C for 10 min. The correctly sized band was excised from the agarose gel and then purified using the DNA Purification Kit (Tiangen, Beijing, China). The PCR products were sequenced by means of the BigDye Terminator Sequencing Kit (ABI, USA) in an ABI 3730xl DNA analyzer, with sequencing primers being provided in Supplementary Figures 1-4.

**EXON 1**

Exon1_F 5'-CTCTCTGGATCCTCGTGAGGTA-3'

Exon1_R 5'-GGTAAGAATGGAAGGGCATTTGTC-3'

PCR_exon1_F 5'-GAGTGGCCAAACATAGCCAG-3'

PCR_exon1_R 5'-GCATTTGTCGGCCAGGAACG-3'

GAGTGGCCAAACATAGCCAGGCTGATGCAAGACCCTGGGAAGAGGAAAGCTGCAGGTGTGTTTGTGCTGGGAGGAGTGGTGACCCTCACCTCACAGTCACCTC**CTCTCTGGATCCTCGTGAGGTA**TAAAGACGAGTCCTCCACCACCAGTCAGGCACACTCTACCACCATGAATCCACTCCTGATCCTTACCTTTGTGGCAGCTGCTCGTGAGTATCATGCCCTGCCTCAGGCCCCAACCACCCCCCCGTTCCTGGCC**GACAAATGCCCTTCCATTCTTACC**

**EXON 2**

Exon2_F1 5'-GTGCTTGTTAAGGATTTCTAATTAGCAGAA-3'

Exon2_R1 5'-ACAGAAGTGGTAGCCAGAATTCAG-3'

Exon2_F2 5'-GCTACAACTGTGAGGAGAATTCTGTC-3'

Exon2_R2 5'-CATCTTACCCAACCTCAGTAGTTCC-3'

PCR_exon2_F 5'-CGCCACCCCTAACATGCTAT-3'

PCR_exon2_R 5'-CTCTCCCAGGCAGACTGGCC-3'

**GTGCTTGTTAAGGATTTCTAATTAGCAGAA**AGCAATCACAGGCTGGGAGCGCCACCCCTAACATGCTATTGACTTGCCTTCTCCCTTCCCATCTCCACTCCAGTTGCTGCCCCCTTTGATGATGATGACAAGATCGTTGGGG**GCTACAACTGTGAGGAGAATTCTGTC**CCCTACCAGGTGTCC**CTGAATTCTGGCTACCACTTCTGT**GGTGGCTCCCTCATCAACGAACAGTGGGTGGTATCAGCAGGCCACTGCTACAAGTCGTAAGTGTGGGGCCCCCGACTGCAAAGCTCCCGGCCAGTCTGCCTGGGAGAGCTTGGCTTCAGCCCAG**GGAACTACTGAGGTTGGGTAAGATG**

*(to be continued)*

**Supplementary Figure 1.** Sequence information pertaining to the targeted sequencing of the *PRSS1* gene. The sequences of the eight primer pairs for targeted sequencing of the *PRSS1* are provided, followed by an indication of their respective locations (underlining) within the *PRSS1* genomic sequence, in the context of the five exons. Coding sequences are highlighted in blue. The sequences of the primer pairs (forward in red, reverse in green) pertaining to the validation of targeted sequencing-derived variants by Sanger sequencing are also provided. These primers were used for both amplifying the different exons and sequencing the resulting PCR fragments. The *PRSS1* genomic sequence was obtained from human GRCh37/hg19 (https://genome.ucsc.edu/). The GenBank (http://www.ncbi.nlm.nih.gov/genbank/) sequence accession number NM_002769.4 was used as the *PRSS1* mRNA reference sequence.

**EXON 3**

Exon3_F1 5'-CATGAGCAGAGAGCTTGAGGAA-3'

Exon3_R1 5'-CGTGAGGAGAGCTTGATTAACATGA-3'

Exon3_F2 5'-CCCAATACGACAGGAAGACTCT-3'

Exon3_R2 5'-GCCAGAGCTCGCAGTGTT-3'

Exon3_F3 5'-GGCACGAAGTGCCTCATCT-3'

Exon3_R3 5'-CCTTGATAGTTTGCATCTCTCTGGT-3'

PCR_exon3_F 5'-AAGGTGGGATAGGTGCCCTG-3'

PCR_exon3_R 5'-GGATGGAGGGAAGTAGAAGGACT-3'

**CATGAGCAGAGAGCTTGAGGAA**CCTGGGGAAGGTGGGATAGGTGCCCTGGCTGTGGGAGAAGGTCTTCACCATGCCTGCCCTGCCCATCAGCCGCATCCAGGTGAGACTGGGAGAGCACAACATCGAAGTCCTGGAGGGGAATGAGCAGTTCATCAATGCAGCCAAGATCATCCGCCACC**CCCAATACGACAGGAAGACTCT**GAACAATGACA**TCATGTTAATCAAGCTCTCCTCACG**TGCAGTAATCAACGCCCGCGTGTCCACCATCTCTCTGCCCACCGCCCCTCCAGCCACT**GGCACGAAGTGCCTCATCT**CTGGCTGGGGC**AACACTGCGAGCTCTGGC**GGTGAGTGGGACCCTTAGTCCTTCTACTTCCCTCCATCCTCACAATTTCCAGAACAAACCATGCCCCTTAACTTGAATCCTCTCACCTCCAGGCTTAAGACACATTTCGAGTGCCCATTACACACAGACTCTGCACTGGGC**ACCAGAGAGATGCAAACTATCAAGG**

**EXON 4**

Exon4_F1 5'-CCTGACCCACATTTCTACTTCCTTTG-3'

Exon4_R1 5'- ACCTTTTGAGTTCAAATCCTTTTCCCT-3'

PCR_exon4_F 5'-GACCCACATTTCTACTTCCTTTGATC-3'

PCR_exon4_R 5'-CTCAGCATGGGAAGGGTTGG-3'

**CCTGACCCACATTTCTACTTCCTTTG**ATCTCTTCCTGATCCTCACAGCCGACTACCCAGACGAGCTGCAGTGCCTGGATGCTCCTGTGCTGAGCCAGGCTAAGTGTGAAGCCTCCTACCCTGGAAAGATTACCAGCAACATGTTCTGTGTGGGCTTCCTTGAGGGAGGCAAGGATTCATGTCAGGTGATTTGACCAACCCTTCCCATGCTGAGGCTCCCACTGATACCTAGGCCCCACC**AGGGAAAAGGATTTGAACTCAAAAGGT**

**EXON 5**

Exon5_F1 5'-GCTATATTCCTCCTCCATCTCTCCATAC-3'

Exon5_R1 5'-GTGAGAACAGGGTCACTTTATTGGTATAG-3'

PCR_exon5_F 5'-TATTCCTCCTCCATCTCTCCATAC-3'

PCR_exon5_R 5'-CAGTGTGAAGGAGTGAGAGG-3'

**GCTATATTCCTCCTCCATCTCTCCATAC**AACTTGTCCCTTCTTCCCCCCAGGGTGATTCTGGTGGCCCTGTGGTCTGCAATGGACAGCTCCAAGGAGTTGTCTCCTGGGGTGATGGCTGTGCCCAGAAGAACAAGCCTGGAGTCTACACCAAGGTCTACAACTATGTGAAATGGATTAAGAACACCATAGCTGCCAATAGCTAAAGCCCCCAGTATCTCTTCAGTCT**CTATACCAATAAAGTGACCCTGTTCTCAC**TGTCTGTGTCTGTGCCTGCTCCCTCTCACTCCTTCACACTG

Supplementary Figure 1 *(continued)*

**EXON 1**

Exon1_F 5'-GGGAGATCTGTGATATAGCCCAGTA-3'

Exon1_R 5'-GTCTAGAAGATAATGTGCTTCACAAAGC-3'

PCR_exon1_F 5'-CCAGGCTATGACACAGAGTC-3'

PCR_exon1_R 5'-GTGCTTCACAAAGCAACAGGTC-3'

CCAGGCTATGACACAGAGTCAATCAATAACCA**GGGAGATCTGTGATATAGCCCAGTA**GGTGGGGCCTTGCTGCCATCTGCCATATGACCCTTCCAGTCCCAGGCTTCTGAAGAGACGTGGTAAGTGCGGTGCAGTTTTCAACTGACCTCTGGACGCAGAACTTCAGCCATGAAGGTAACAGGCATCTTTCTTCTCAGTGCCTTGGCCCTGTTGAGTCTATCTGGTAAGTGTTGCATATTTTTCAAATTTAAATAAAACTGTTTTGACCTGTT**GCTTTGTGAAGCACATTATCTTCTAGAC**

**EXON 2**

Exon2_F 5'-GGGAATGAAAGAGCCTAGTAAAGAAGT-3'

Exon2_R 5'-CCCTCCCTAGCATTCATACTCCT-3'

PCR_exon2_F 5'- GGGTGGGGAATGAAAGAGCC-3'

PCR_exon2_R 5'-AAAGGTGACAGCAAGGCTGC-3'

GGGTG**GGGAATGAAAGAGCCTAGTAAAGAAGT**CACAGTCTGCAATGAAAGCAGAGAATTCTGATGAAGAATAGATCTGACTTCTTTCATTTAGGACCCAACTTACCATATCTGATTTATTTCTAGGTAACACTGGAGCTGACTCCCTGGGAAGAGAGGTAAAGAGATATTTGTAATTTCTTATTTCTCAGACTGGAACAGTTTGATCCAACAAAAATGCAGCCTTGCTGTCACCTTTCAGTTTAGCCTGAAGTTAAG**AGGAGTATGAATGCTAGGGAGGG**

*(to be continued)*

**Supplementary Figure 2**. Sequence information pertaining to the targeted sequencing of the *SPINK1* gene. The sequences of the four primer pairs for targeted sequencing of the *SPINK1* gene are first provided, followed by an indication of their respective locations (underlining) within the *SPINK1* genomic sequence, in the context of the four exons. Coding sequences are highlighted in blue. The sequences of the primer pairs (forward in red, reverse in green) pertaining to the validation of targeted sequencing-derived variants by Sanger sequencing are also provided. These primers were used for both amplifying the different exons and sequencing the resulting PCR fragments. The *SPINK1* genomic sequence was obtained from human GRCh37/hg19 (https://genome.ucsc.edu/). The GenBank (http://www.ncbi.nlm.nih.gov/genbank/) sequence accession number NM_003122.4 was used as the *SPINK1* mRNA reference sequence.

**EXON 3**

Exon3_F 5'-ACCATTTCAGAGATTTTGCTATGAACTCA-3'

Exon3_R 5'-GGGTGAGATTCATATTATCAGTACACTTGA-3'

PCR_exon3_F 5'-CCAATCACAGTTATTCCCCAGAG-3'

PCR_exon3_R 5'-GTTTGCTTTTCTCGGGGTGAG-3'

CCAATCACAGTTATTCCCCAGAGAAATAAA**ACCATTTCAGAGATTTTGCTATGAACTCA**AGAATGGAGAATAATGGGAAATGATTCTGTTTAATTCCATTTTTAGGCCAAATGTTACAATGAACTTAATGGATGCACCAAGATATATGACCCTGTCTGTGGGACTGATGGAAATACTTATCCCAATGAATGCGTGTTATGTTTTGAAAATCGGTGAGTACAAACTTGAGTTTCTTTTAAACTATATATTTTAAGTTAGTTATCT**TCAAGTGTACTGATAATATGAATCTCACCC**CGAGAAAAGCAAAC

**EXON 4**

Exon4_F 5'-TCAAACCTCTCCAACTTTAAATGAAGCT-3'

Exon4_R 5'-CATACCTTTGAGGAAAAAGCAAATGGA-3'

PCR_exon4_F 5'-CCCTGTTTTTCTCCCATAGTCAC-3'

PCR_exon4_R 5'-CCAAAGTCCCCTGACCCTGG-3'

**TCAAACCTCTCCAACTTTAAATGAAGCT**GTTATTTTTCCCCCTGTTTTTCTCCCATAGTCACTTTTTCATCAGTGAAGTTTAAGCTGATATATTTTTTTTAATCTCTACTGCAGGAAACGCCAGACTTCTATCCTCATTCAAAAATCTGGGCCTTGCTGAGAACCAAGGTTTTGAAATCCCATCAGGTCACCGCGAGGCCTGACTGGCCTTATTGTTGAATAAATGTATCTGAATATCCCCTGTTGTT**TCCATTTGCTTTTTCCTCAAAGGTATG**TTTGATTATACCAGGGTCAGGGGACTTTGG

Supplementary Figure 2 *(continued)*

**EXON 1**

Exon1_F 5'-ACTCACCTGCTCCTGCCTATAA-3'

Exon1_R 5'-AGCTGGTTGGTAGCATCTGAAC-3'

PCR_exon1_F 5'-CTGTCTCCTGCCCAAATTAAAC-3'

PCR_exon1_R 5'-CGATGGTCCCTGGGGTGAAC-3'

CTGTCTCCTGCCCAAATTAAACAGTAACCACCCAAGGTCAGGGAAAGGGCCTTATCAGAGCAGGTTTCTCTCCAAGGGGCGGTTTGGCCTTGACCAGGTGC**ACTCACCTGCTCCTGCCTATAA**GTGTGCCCCAGCCCATCCCGATGGTCAGCCAGTCCTGAGCACCTAACCATGTTGGGCATCACTGTCCTCGCTGCGCTCTTGGCCTGTGGTAAGCGGTGGGGTGGGGCTGCAGCTAGCAGGCTGTGAGCTCGGGCTGGCCTCCAGGGCAAGGACGGGATGGGGAGTGGGGGGGCCTCTGCTCTCCAGGTAAGACACTTTGGGGTCCCCTGTGG**GTTCAGATGCTACCAACCAGCT**GTGTGTCAGACAAGCCACAGCCTGCCTGCCAACTCTGTTTCCACATCTGCAAAACAGGGAAGATGATCTGACGTGTCCCCAGTTAGAAATGAGGTGTGGAAAGCACCACGCAAGGGGGAGGGACACGAGATGCCTCATCCTGGGTTCACCCCAGGGACCATCG

**EXON 2 AND EXON 3**

Exon2_F 5'-CTACCAGCCCTATTCACTGGTT-3'

Exon2_R 5'-CTTGAGGTACTGGAGGGAGATCT-3'

Exon3_F 5'-GTAGGGCTGGGAGGTACAGATA-3'

Exon3_R 5'-GCGGAGAAGCTGCTCAGAAAAA-3'

PCR_exon2-3_F 5'-CCCCGTGACACAGTAAAATATC-3'

PCR_exon2-3_R 5'-TGGGTGTGAGTAGATTATGTAG-3'

CCCCGTGACACAGTAAAATATCAACCCCGTGTCTGCCCAGCCCCAACTCTGTGCTTCTTCCACCTGCCCACCCTCCCACCCCTTTCCCCGTGGG**CTACCAGCCCTATTCACTGGTT**CTTCTGGCCTCCTGTCTCCCCAGCCTCCAGCTGTGGGGTGCCCAGCTTCCCGCCCAACCTATCCGCCCGAGTGGTGGGAGGAGAGGATGCCCGGCCCCACAGCTGGCCCTGGCAGGTAAGCCTGT**GTAGGGCTGGGAGGTACAGATA**GAGAGGGTGGCGGGGTGAGGGTCCCAGGGACCTGCAGGCTGACACACAGCCCTCCCCACCCTCCTGCAGATCTCCCTCCAGTACCTCAAGAACGACACGTGGAGGCATACGTGTGGCGGGACTTTGATTGCTAGCAACTTCGTCCTCACTGCCGCCCACTGCATCAGGTGTGCGGGGATGATACCCTGAGACCTGGCCATCGTCCGGGGGCGGAAGCCTGATACTCTGC**TTTTTCTGAGCAGCTTCTCCGC**ACTCCAGGCACTGGGCTACATAATCTACTCACACCCA

*(to be continued)*

**Supplementary Figure 3.** Sequence information pertaining to the targeted sequencing of the *CTRC* gene. The sequences of the 12 primer pairs for targeted sequencing of the *CTRC* gene are first provided, followed by an indication of their respective locations (underlining) within the *CTRC* genomic sequence, in the context of the eight exons. Coding sequences are highlighted in blue. The sequences of the primer pairs (forward in red, reverse in green) pertaining to the validation of targeted sequencing-derived variants by Sanger sequencing are also provided. These primers were used for both amplifying the different exons and sequencing the resulting PCR fragments. The *CTRC* genomic sequence was obtained from human GRCh37/hg19 (https://genome.ucsc.edu/). The GenBank (http://www.ncbi.nlm.nih.gov/genbank/) sequence accession number NM_007272.2 was used as the *CTRC* mRNA reference sequence.

**EXON 4**

Exon4_F1 5'-GAGCAGCAAAGTCACAACATGA-3'

Exon4_R1 5'-CCCACAAACAGGGATCCTTCTT-3'

Exon4_F2 5'-GAAAGAACAACCTGGAGGTGGAA-3'

Exon4_R2 5'-ACAGTCATTTGCTGGCTTTCCA-3'

PCR_exon4_F 5'-AAGGACAATGGGAACACTCTCT-3'

PCR_exon4_R 5'-TCAGGTATGGGGTGCGACAG-3'

**GAGCAGCAAAGTCACAACATGA**ACCCAAGTCCCTTGACCCCAAGTCCCAGGCCCTTCCCCTCACCCTGGGAAAGGACAATGGGAACACTCTCTTCCCCCAAAATGAGTCCCACTAAAGCCCCGAGCTCCCTCTCTATCCCACTGGCTGGCAGGACCAGGGGGCCACCCTGACCTGGACCCCTTCCTCTGCCCAGCAACACCCGGACCTACCGTGTGGCCGTGG**GAAAGAACAACCTGGAGGTGGAA**GACG**AAGAAGGATCCCTGTTTGTGGG**TGTGGACACCATCCACGTCCACAAGAGATGGAATGCCCTCCTGTTGCGGTGAGTGACAGACTGCCCATCCCACAGCCACTGGGGGCAGTGTGGAAGGAGGGGTCCCCAAGACGGAGCCGCAGAGCCTGTCGCACCCCATACCTGACACCCCATCCTCACCC**TGGAAAGCCAGCAAATGACTGT**

**EXON 5**

Exon5_F1 5'-GTTAACCCTTTCCCGAGGTGAT-3'

Exon5_R1 5'-TGCAAGCTTGATGAGGGCAATA-3'

Exon5_F2 5'-ACTCACCCTCTCCACTTTGGAT-3'

Exon5_R2 5'-TTCAGGAAGGCTCTCAGGATTTG-3'

PCR_exon5_F 5'-CAGTGCGGGTGATCATGTTAGA-3'

PCR_exon5_R 5'-TCAGAGACCCTTGCTAGAGTG-3'

**GTTAACCCTTTCCCGAGGTGAT**GACATGTGAGAAGAAGCTATGTCAACAGTGCGGGTGATCATGTTAGATGGTGAATCGTTTGTCCTGATATTCCCAAAGGCCAAGAAGAAGCTGGCAGTCAGGATGTTTGTGAAGGACCCCTGAGCACCCTGGGCCTGACTCCCAACTCACAGCCCGAGCCCCTTAGCCTGAGCTTGTGGGGCCAGGGCCTCCTGCCC**ACTCACCCTCTCCACTTTGGAT**TCCAGCAATGA**TATTGCCCTCATCAAGCTTGCA**GAGCATGTGGAGCTGAGTGACACCATCCAGGTGGCCTGCCTGCCAGAGAAGGACTCCCTGCTCCCCAAGGACTACCCCTGCTATGTCACCGGCTGGGGCCGCCTCTGGAGTGAGTATCGTCCCTGG**CAAATCCTGAGAGCCTTCCTGAA**GGAAGCAGGACGTCACCCACATTTGTCCACCACTTTGCCTTTTTGCCTTCACATTTTCATGTCTTTGTAAACTTTGTAACAATAACAGCTAATATTTACTAAGCACATACCATGTGACAGACACTCTAGCAAGGGTCTCTGA

**EXON 6**

Exon6_F1 5'-CCTTGGCTTGCCTCTTGATGAA-3'

Exon6_R1 5'-GCACACCATGGTTTTCTTCACC-3'

Exon6_F2 5'-CCACGTGCTCCAGGATTGAC-3'

Exon6_R2 5'-CCACTTGGCCACTTGGAGTAG-3'

PCR_exon6_F 5'-TTGATGAAGGGCAGGTGTGTG-3'

PCR_exon6_R 5'-TGGGGCTTGAACTCAGATACTC-3'

Supplementary Figure 3 *(continued)*

**CCTTGGCTTGCCTCTTGATGAA**GGGCAGGTGTGTGGTCCGCACACTGTCTCAGCCGGCGCTCCCCTGGGTCCTGTCCCAGGCATCTGCTCCCTGAAGGCCTGGGGAGGGGCTGGACTGGGCTTCCCGGCTGCCTCCCTGGTCACTGCTCACTCTCTCCCCAGCCAACGGCCCCATTGCTGATAAGCTGCAGCAGGGCCTGCAGCCCGTGGTGGATCACG**CCACGTGCTCCAGGATTGAC**TGGTGGGGCTTCAG**GGTGAAGAAAACCATGGTGTGC**GCTGGGGGCGATGGCGTCATCTCAGCCTGCAATGTGAGTGGCTAGGTTCTGCACCTTGTCC**CTACTCCAAGTGGCCAAGTGG**ATGTGGGCAAAGGGGGGTGCGATGGACCAAACCCTTCTCCTGGGAGGAGACTGAGCGAGCCCTGGCTGGGCCCAGCAGGCTCAGGGTAAGCCCATCACCCCCCATCACTGTGGTGAGAGCATGAGCATTGAGCACGAGTATCTGAGTTCAAGCCCCA

**EXON 7**

Exon7_F1 5'-ATCTGTCCACTAACTAAGGCTGAGA-3'

Exon7_R1 5'-CCTCGTTGATCCAGTCGATGTAG-3'

Exon7_F2 5'-GCAAGAAGCCGGTAGTCTACAC-3'

Exon7_R2 5'-AATAAACGCATGAATGAGTGAATGAGTG-3'

PCR_exon7_F 5'-CCAAATCTGTCCACTAACTAAG-3'

PCR_exon7_R 5'-CACTGAATGAGCAAATGAATGAG-3'

CCAA**ATCTGTCCACTAACTAAGGCTGAGA**AGCCAACCCACATCCCCCACCCCAACCCAGTCCTGCTTCCCAAGACTTCCTCTGGGGGGGGGCCTGGTGGCTTATGCCCTCCCGGTCTGGTGCAGGGGGACTCCGGTGGCCCACTGAACTGCCAGTTGGAGAACGGTTCCTGGGAGGTGTTTGGCATCGTCAGCTTTGGCTCCCGGCGGGGCTGCAACACCC**GCAAGAAGCCGGTAGTCTACAC**CCGGGTGTCCGC**CTACATCGACTGGATCAACGAGG**TGGGTGCTGCCTCCACAGCTGTCCCTGCACCTGTCAGCCCCTCCCCCTCACTCACCCATCCCCT**CACTCATTCACTCATTCATGCGTTTATT**CATTCATTCATTTATTCACTCATTCATGCATTTATTCACTCATTCATGCATTCATTCATTTATTCACTTATTCAGTCACTCATTCATGTATTTATTCATTTATTCATTCACTCATGCATTCATTCATTCATTTATTCACTTATTCATTCACTCATTCATGTATTCATTCATTCATGCATTTATTTACTCATTCATCCATTTATTCACTCATTCATTTGCTCATTCAGTG

**EXON 8**

Exon8_F 5'-GTGTGTTCCAAGACCCATTTGC-3'

Exon8_R 5'-GCCCGAGGAGAAGGAAGTTTAT-3'

PCR_exon8_F 5'-CTTGAGAGTAGGGGAACAGAG-3'

PCR_exon8_R 5'-GCGTCCTTCACTCCCATCCC-3'

CTTGAGAGTAGGGGAACAGAGGGTCACCCTGGGCTGGGGGCTTCCCCATTGGGGACAGA**GTGTGTTCCAAGACCCATTTGC**ACAGTGGCCTGAAATGCTGAGGGCCTCAGACCCCTTGAACAGGGACAAGGCTGGCATGTGAAGGCCGGGGGCTGCTGGCCATGCCCCCATGGACCCACCCTCCGGGCAGAGCCCTGTGCCACCCTAGAAGGTGGCACAGCCCTGAGTCTCTCACACTGTTCTCTGCTCCTCCAGAAAATGCAGCTGTGATTTGTTGCTGGGAGCGGCGGCAGCGAGTCCCTGCAACAGCA**ATAAACTTCCTTCTCCTCGGGC**CACCTGGATCCTTGATTTGTGCAGCTTCTGTTGCTTCCCTCCTCTCTGGTGCTGCCCCTTTCCACACTATGGAGCCAAAGAGAGACCCCACTCAGCCAGTTTCCCCCACCCTGCATTAGACAGGTGGGGAAACAGAGGCCGGGAGAGAGGGCCAAGGAAGGAGCCTCCTGGGGCATTAATGGGAGGCAGGGGGCTGGGGTGGAGAGCCCAGGGAGTCCTGCGTGAAGCCGGAGGGGATGGGAGTGAAGGACGC

Supplementary Figure 3 *(continued)*

**EXON 1**

Exon1_F 5'-CAGCAGGTCAGAGAAAAAGGGTT-3'

Exon1_R 5'-GCGCATCTTTTTAAAAACTGCTTATTCC-3'

PCR_exon1_F 5'-CAAAAGGAAGGGGTGGTGTG-3'

PCR_exon1_R 5'-TTACCCCAAACCCAACCCAT-3'

CAAAAGGAAGGGGTGGTGTGCGGAGTAGGGGTGGGTGGGGGGAATTGGAAGCAAATGACATCA**CAGCAGGTCAGAGAAAAAGGGTT**GAGCGGCAGGCACCCAGAGTAGTAGGTCTTTGGCATTAGGAGCTTGAGCCCAGACGGCCCTAGCAGGGACCCCAGCGCCCGAGAGACCATGCAGAGGTCGCCTCTGGAAAAGGCCAGCGTTGTCTCCAAACTTTTTTTCAGGTGAGAAGGTGGCCAACCGAGCTTCGGAAAGACACGTGCCCACGAAAGAGGAGGGCGTGTGTATGGGTTGGGTTTGGGGTAAA**GGAATAAGCAGTTTTTAAAAAGATGCGC**

**EXON 2**

Exon2_F1 5'-CATACTATTATTCCCTCCCAATCCCTTT-3'

Exon2_R1 5'-ATCCTTTCCTCAAAATTGGTCTGGT-3'

Exon2_F2 5'-TCAAGTGAATATCTGTTCCTCCTCTCTTT-3'

Exon2_R2 5'-GCCACCATACTTGGCTCCTATTTTTAAATA-3'

PCR_exon2_F 5'-TGTAAGAGATGAAGCCTGGTATT-3'

PCR_exon2_R 5'-CATGCACTACCATTCCCAGC-3'

**CATACTATTATTCCCTCCCAATCCCTTT**GACAAAGTGACAGTCACATTAGTTCAGAGATATTGATGTTTTATACAGGTGTAGCCTGTAAGAGATGAAGCCTGGTATTTATAGAAATTGACTTATTTTATTCTCATATTTACATGTGCATAATTTTCCATATGCCAGAAAAGTTGAATAGTATCAGATTCCAAATCTGTATGGAGACCAAA**TCAAGTGAATATCTGTTCCTCCTCTCTTT**ATTTTAGCTGG**ACCAGACCAATTTTGAGGAAAGGAT**ACAGACAGCGCCTGGAATTGTCAGACATATACCAAATCCCTTCTGTTGATTCTGCTGACAATCTATCTGAAAAATTGGAAAGGTATGTTCATGTACATTGTTTAGTTGAAGAGAGAAATTCATATTATTAATTATTTAGAGAAGAGAAAGCAAACATATTATAAGTTTAATTCTTA**TATTTAAAAATAGGAGCCAAGTATGGTGGC**TAATGCCTGTAATCCCAACTATTTGGGAGGCCAAGATGAGAGGATTGCTTGAGACCAGGAGTTTGATACCAGCCTGGGCAACATAGCAAGATGTTATCTCTACACAAAATAAAAAAGTTAGCTGGGAATGGTAGTGCATG

*(to be continued)*

**Supplementary Figure 4.** Sequence information pertaining to the targeted sequencing of the *CFTR* gene. The sequences of the 49 primer pairs for targeted sequencing of the *CFTR* gene are first provided, followed by an indication of their respective locations (underlining) within the *CFTR* genomic sequence, in the context of the 27 exons. Coding sequences are highlighted in blue. The sequences of the primer pairs (forward in red, reverse in green) pertaining to the validation of targeted sequencing-derived variants by Sanger sequencing are also provided. These primers were used for both amplifying the different exons and sequencing the resulting PCR fragments. The *CFTR* genomic sequence was obtained from human GRCh37/hg19 (https://genome.ucsc.edu/). The GenBank (http://www.ncbi.nlm.nih.gov/genbank/) sequence accession number NM_000492.3 was used as the *CFTR* mRNA reference sequence.

**EXON 3**

Exon3_F 5'-TAGGACAACTAAAATATTTGCACATGCAAC-3'

Exon3_R 5'-ACCAGATTTCGTAGTCTTTTCATAATCACA-3'

PCR_exon3_F 5'-GGCTGAGTGTTTGGTGTTGT-3'

PCR_exon3_R 5'-TAAATTGCCACCCGTGTTCC-3'

GGCTGAGTGTTTGGTGTTGTATGGTCTCCATGAGATTTTGTCTCTATAATACTTGGGTTAATCTCCTTGGATATACTTGTGTGAATCAAACTATGTTAAGGGAAA**TAGGACAACTAAAATATTTGCACATGCAAC**TTATTGGTCCCACTTTTTATTCTTTTGCAGAGAATGGGATAGAGAGCTGGCTTCAAAGAAAAATCCTAAACTCATTAATGCCCTTCGGCGATGTTTTTTCTGGAGATTTATGTTCTATGGAATCTTTTTATATTTAGGGGTAAGGATCTCATTTGTACATTCATTATGTATCACATAACTATATTCATTTT**TGTGATTATGAAAAGACTACGAAATCTGGT**GAATAGGTGTAAAAATATAAAGGATGAATCCAACTCCAAACACTAAGAAACCACCTAAAACTCTAGTAAGGATAAGTAAAAATCCTTTGGAACTAAAATGTCCTGGAACACGGGTGGCAATTTA

**EXON 4**

Exon4_F1 5'-AAGTCTCCTCTAAAGATGAAAAGTCTTGTG-3'

Exon4_R1 5'-CCTATGCCTAGATAAATCGCGATAGAG-3'

Exon4_F2 5'-CATAGCTTCCTATGACCCGGATAAC-3'

Exon4_R2 5'-GTACCAGCTCACTACCTAATTTATGACATT-3'

PCR_exon4_F 5'-ACTTGTCTCCCACTGTTGCT-3'

PCR_exon4_R 5'-CAGGTAGTGAGCTGGTACAAGT-3'

ACTTGTCTCCCACTGTTGCTATAACAAATCCCAAGTCTTATTTCAAAGTACCAAGATATTGAAAATAGTGCTAAGAGTTTCACATATGGTATGACCCTCTATATAAACTCATTTT**AAGTCTCCTCTAAAGATGAAAAGTCTTGTG**TTGAAATTCTCAGGGTATTTTATGAGAAATAAATGAAATTTAATTTCTCTGTTTTTCCCCTTTTGTAGGAAGTCACCAAAGCAGTACAGCCTCTCTTACTGGGAAGAAT**CATAGCTTCCTATGACCCGGATAAC**AAGGAGGAACG**CTCTATCGCGATTTATCTAGGCATAGG**CTTATGCCTTCTCTTTATTGTGAGGACACTGCTCCTACACCCAGCCATTTTTGGCCTTCATCACATTGGAATGCAGATGAGAATAGCTATGTTTAGTTTGATTTATAAGAAGGTAATACTTCCTTGCACAGGCCCCATGGCACATATATTCTGTATCGTACATGTTTT**AATGTCATAAATTAGGTAGTGAGCTGGTAC**AAGT

**EXON 5**

Exon5_F1 5'-CTAGAAGCATGCCAGTATAATATTGACTGT-3'

Exon5_R1 5'-GAAAGGAGACTAACAAGTTGTCCAATACTT-3'

Exon5_F2 5'-CTTTAAAGCTGTCAAGCCGTGTT-3'

Exon5_R2 5'-TCCGCCTTTCCAGTTGTATAATTTATAACA-3'

PCR_exon5_F 5'-CCCGCACAATATCAATGGGT-3'

PCR_exon5_R 5'-ACTCCGCCTTTCCAGTTGTA-3'

CCCGCACAATATCAATGGGTATTTAAGTATAATATCATTCTCATTGTGAGGAGAAAAAATAATTATTTCTGCCTAGATGCTGGGAAATAAAACAA**CTAGAAGCATGCCAGTATAATATTGACTGT**TGAAAGAAACATTTATGAACCTGAGAAGATAGTAAGCTAGATGAATAGAATATAATTTTCATTACCTTTACTTAATAATGAATGCATAATAACTGAATTAGTCATATTATAATTTTACTTATAATATATTTGTATTTTGTTTGTTGAAATTATCTAACTTTCCATTTTTCTTTTAG**ACTTTAAAGCTGTCAAGCCGTGTT**CTAGATAAAAT**AAGTATTGGACAACTTGTTAGTCTCCTTTC**CAACAACCTGAACAAATTTGATGAAGTATGTACCTATTGATTTAATCTTTTAGGCACTAT**TGTTATAAATTATACAACTGGAAAGGCGGA**GT

Supplementary Figure 4 *(continued)*

**EXON 6**

Exon6_F 5'-GACACCTGTTTTTGCTGTGCTT-3'

Exon6_R 5'-CTGGTTTTACTAAAGTGGGCTTTTTGAAA-3'

PCR_exon6_F 5'-AGGGGTGGAAGATACAATGACA-3'

PCR_exon6_R 5'-CGCCTCTAATCCCAGCTACT-3'

AGGGGTGGAAGATACAAT**GACACCTGTTTTTGCTGTGCTT**TTATTTTCCAGGGACTTGCATTGGCACATTTCGTGTGGATCGCTCCTTTGCAAGTGGCACTCCTCATGGGGCTAATCTGGGAGTTGTTACAGGCGTCTGCCTTCTGTGGACTTGGTTTCCTGATAGTCCTTGCCCTTTTTCAGGCTGGGCTAGGGAGAATGATGATGAAGTACAGGTAGCAACCTATTTTCATAACTTGAAAGTTTTAAAAATTATGT**TTTCAAAAAGCCCACTTTAGTAAAACCAG**GACTGCTCTATGCATAGAACAGTGATCTTCAGTGTCATTAAATTTTTTTTTTTTTTTTTTTTTTGAGACAGAGTCTAGATCTGTCACCCAGGCTGGAGTGCAGTGGCACGATCTTGGCTCACTGCACTGCAACTTCTGCCTCCCAGGCTCAAGCAATTCTCCTGCCTCAGCCTCCGGAGTAGCTGGGATTAGAGGCG

**EXON 7**

Exon7_F1 5'-GGATAGAGATAGCATATGGAATGAGTCTG-3'

Exon7_R1 5'-ATCATTTCTGAGGTAATCACAAGTCTTTCA-3'

Exon7_F2 5'-GATTGATTGATTTACAGAGATCAGAGAGCT-3'

Exon7_R2 5'-TATGAGGTGGAAGTCTACCATGATAAACAT-3'

PCR_exon7_F 5'-AGATACCCACCGCTCATAGG-3'

PCR_exon7_R 5'-AACACCCTGGACCAACTACA-3'

AGATACCCACCGCTCATAGGCTGTCATAAG**GGATAGAGATAGCATATGGAATGAGTCTG**TACAGCGTCTGGCACATAGGAGGCATTTACCAAACAGTAGTTATTATTTTTGTTACCATCTATTTGATAATAAAATAATGCCCATCTGTTGAATAAAAGAAATATGACTTAAAACCTTGAGCAGTTCTTAATAGATAATTTGACTTGTTTTTACTATTAGATTGATTGATTGATT**GATTGATTGATTTACAGAGATCAGAGAGCT**GGGAAGATCAG**TGAAAGACTTGTGATTACCTCAGAAATGAT**TGAAAATATCCAATCTGTTA**AG**GCATACTGCTG**G**G**A**AGAAGCAATGGAAAAAATGATTGAAAACTTAAGACAGTAAGTTGTTCCAATAATTTCAATATTGTTAGTAATTCTGTCCTTAATTTTTTAAAAAT**ATGTTTATCATGGTAGACTTCCACCTCATA**TTTGATGTTTGTGACAATCAAATGATTGCATTTAAGTTCTGTCAATATTCATGCATTAGTTGCACAAATTCACTTTCATGGGCTGTAGTTTTATGTAGTTGGTCCAGGGTGTT

**EXON 8**

Exon8_F1 5'-CCAAGGTCACACAGGTCATATGAT-3'

Exon8_R1 5'-CCTTTGATTAGTGCATAGGGAAGCA-3'

Exon8_F2 5'-CTTCTTCTCAGGGTTCTTTGTGGT-3'

Exon8_R2 5'-GCACATTTTTGCAAAGTTCATTAGAACTGA-3'

PCR_exon8_F 5'-CCCAAGGTCACACAGGTCAT-3'

PCR_exon8_R 5'-TGCCACTCTCATCCATCATACT-3'

C**CCAAGGTCACACAGGTCATATGAT**GTGGAGCCAGGTTAAAAATATAGGCAGAAAGACTCTAGAGACCATGCTCAGATCTTCCATTCCAAGATCCCTGATATTTGAAAAATAAAATAACATCCTGAATTTTATTGTTATTGTTTTTTATAGAACAGAACTGAAACTGACTCGGAAGGCAGCCTATGTGAGATACTTCAATAGCTCAGCCTT**CTTCTTCTCAGGGTTCTTTGTGGT**GTTTTTATCTG**TGCTTCCCTATGCACTAATCAAAGG**AATCATCCTCCGGAAAATATTCACCACCATCTCATTCTGCATTGTTCTGCGCATGGCGGTCACTCGGCAATTTCCCTGGGCTGTACAAACATGGTATGACTCTCTTGGAGCAATAAACAAAATACAGGTAATGTACCATAATGCTGCATTATATACTATGATTTAAATAATCAGTCAATAGA**TCAGTTCTAATGAACTTTGCAAAAATGTGC**GAAAAGATAGAAAAAGAAATTTCCTTCACTAGGAAGTTATAAAAGTTGCCAGCTAATACTAGGAATGTTCACCTTAAACTTTTCCTAGCATTTCTCTGGACAGTATGATGGATGAGAGTGGCA

Supplementary Figure 4 *(continued)*

**EXON 9**

Exon9_F1 5'-ATCCTAGTGCTTGGCAAATTAACTTTAGA-3'

Exon9_R1 5'-TCCTCCCAGAAGGCTGTTACAT-3'

Exon9_F2 5'-AACGACTACAGAAGTAGTGATGGAGA-3'

Exon9_R2 5'-GCCATTAGGATGAAATCCATATTCACAAAG-3'

PCR_exon9_F 5'-TGATGAATCCTAGTGCTTGGC-3'

PCR_exon9_R 5'-GCCACCATGCCCAGTTAATT-3'

TGATGA**ATCCTAGTGCTTGGCAAATTAACTTTAGA**ACACTAATAAAATTATTTTATTAAGAAATAATTACTATTTCATTATTAAAATTCATATATAAGATGTAGCACAATGAGAGTATAAAGTAGATGTAATAATGCATTAATGCTATTCTGATTCTATAATATGTTTTTGCTCTCTTTTATAAATAGGATTTCTTACAAAAGCAAGAATATAAGACATTGGAATATAACTT**AACGACTACAGAAGTAGTGATGGAGAATGTAACAGCCTTCTGGGAGGA**GGTCAGAATTTTTAAAAAATTGTTTGCTCTAAACACCTAACTGTTTTCTT**CTTTGTGAATATGGATTTCATCCTAATGGC**GAATAAAATTAGAATGATGATATAACTGGTAGAACTGGAAGGAGGATCACTCACTTATTTTCTAGATTAAGAAGTAGAGGAATGGCCAGGTGCTCATGGTTGTAATCCCAGCACTTTGGGAGACCAAGGCGGGTGGATCACCTGAGGTCAGGAGTTCAAGACCAGCCTGGCCAACATGGTAAAACCCGGTCTCTACTAAAAATACAAAAAATTAACTGGGCATGGTGGC

**EXON 10**

Exon10_F1 5'-AGCATCTATTGAAAATATCTGACAAACTCATCT-3'

Exon10_R1 5'-ACAAAAGAACTACCTTGCCTGCT-3'

Exon10_F2 5'-TTCAAGATAGAAAGAGGACAGTTGTTGG-3'

Exon10_R2 5'-AATACCTTCCAGCACTACAAACTAGAAAAA-3'

PCR_exon10_F 5'-CAGTGTAATGGATCATGGGCC-3'

PCR_exon10_R 5'-ACAGTGTTGAATGTGGTGCA-3'

CAGTGTAATGGATCATGGGCCATGTGCTTTTCAAACTAATTGTACATAAAACA**AGCATCTATTGAAAATATCTGACAAACTCATCT**TTTATTTTTGATGTGTGTGTGTGTGTGTGTGTGTTTTTTTAACAGGGATTTGGGGAATTATTTGAGAAAGCAAAACAAAACAATAACAATAGAAAAACTTCTAATGGTGATGACAGCCTCTTCTTCAGTAATTTCTCACTTCTTGGTACTCCTGTCCTGAAAGATATTAAT**TTCAAGATAGAAAGAGGACAGTTGTTGG**CGGTTGCTGGATCCACTGG**AGCAGGCAAGGTAGTTCTTTTGT**TCTTCACTATTAAGAACTTAATTTGGTGTCCATGTCTCTTTT**TTTTTCTAGTTTGTAGTGCTGGAAGGTATT**TTTGGAGAAATTCTTACATGAGCATTAGGAGAATGTATGGGTGTAGTGTCTTGTATAATAGAAATTGTTCCACTGATAATTTACTCTAGTTTTTTATTTCCTCATATTATTTTCAGTGGCTTTTTCTTCCACATCTTTATATTTTGCACCACATTCAACACTGT

**EXON 11**

Exon11_F1 5'-CTCTTTTACTTTCCCTTGTATCTTTTGTGC-3'

Exon11_R1 5'-TGAAGGCTCCAGTTCTCCCATA-3'

Exon11_F2 5'-TGATGGGTTTTATTTCCAGACTTCACTT-3'

Exon11_R2 5'-GTGAAGGGTTCATATGCATAATCAAAAAGT-3'

PCR_exon11_F 5'-AGAACAGCACTCGACACAGA-3'

PCR_exon11_R 5'-TTGGGTAGTGTGAAGGGTTC-3'

AGAACAGCACTCGACACAGAGTGAGCACTTGGCAACTGTTAGCTGTTACTAACCTTTCCCATTCTTCCTCCAAACCTATTCCAACTATCTGAATCATGTGCCCCTTCTCTGTGAACCTCTATCATAATACTTGTCACACTGTATTGTAATTGT**CTCTTTTACTTTCCCTTGTATCTTTTGTGC**ATAGCAGAGTACCTGAAACAGGAAGTATTTTAAATATTTTGAATCAAATGAGTTAATAGAATCTTTACAAATAAGAATATACACTTCTGCTTAGGATGATAATTGGAGGCAAGTGAATCCTGAGCGTGATTTGATAATGACCTAATAA**TGATGGGTTTTATTTCCAGACTTCACTT**CTAATGGTGAT**TATGGGAGAACTGGAGCCTTCA**GAGGGTAAAATTAAGCACAGTGGAAGAATTTCATTCTGTTCTCAGTTTTCCTGGATTATGCCTGGCACCATTAAAGAAAATATCATCTTTGGTGTTTCCTATGATGAATATAGATACAGAAGCGTCATCAAAGCATGCCAACTAGAAGAGGTAAGAAACTATGTGAAA**ACTTTTTGATTATGCATATGAACCCTTCAC**ACTACCCAA

Supplementary Figure 4 *(continued)*

**EXON 12**

Exon12_F 5'-GTTCAAAATTTCAACTGTGGTTAAAGCAAT-3'

Exon12_R 5'-ACATGAATGACATTTACAGCAAATGCTT-3'

PCR_exon12_F 5'-GGAAGATGTGCCTTTCAAATTCA-3'

PCR_exon12_R 5'-TTGGAGTGGCAGGGTCTATG-3'

**GTTCAAAATTTCAACTGTGGTTAAAGCAAT**AGTGTGATATATGATTACATTAGAAGGAAGATGTGCCTTTCAAATTCAGATTGAGCATACTAAAAGTGACTCTCTAATTTTCTATTTTTGGTAATAGGACATCTCCAAGTTTGCAGAGAAAGACAATATAGTTCTTGGAGAAGGTGGAATCACACTGAGTGGAGGTCAACGAGCAAGAATTTCTTTAGCAAGGTGAATAACTAATTATTGGTCTAGC**AAGCATTTGCTGTAAATGTCATTCATGT**AAAAAAATTACAGACATTTCTCTATTGCTTTATATTCTGTTTCTGGAATTGAAAAAATCCTGGGGTTTTATGGCTAGTGGGTTAAGAATCACATTTAAGAACTATAAATAATGGTATAGTATCCAGATTTGGTAGAGATTATGGTTACTCAGAATCTGTGCCCGTATCTTGGTGTCAGTGTATTTGTTTGCCTCATAGTATAGTTTACTACAAATGGAAAACTCTAGGATTCTGCATAATACTGGACAGAGAAGATGTAAATATCTGTTAGTTCCATCATAGACCCTGCCACTCCAA

**EXON 13**

Exon13_F 5'-GTAATGCATGTAGTGAACTGTTTAAGGC-3'

Exon13_R 5'-TCTCAAGAGGTAAAATGCAATCTATGATGG-3'

PCR_exon13_F 5'-CAGTGAATCGATGTGGTGACC-3'

PCR_exon13_R 5'-CATGAGGCGGTGAGAAAAGG-3'

CAGTGAATCGATGTGGTGACCATATT**GTAATGCATGTAGTGAACTGTTTAAGGC**AAATCATCTACACTAGATGACCAGGAAATAGAGAGGAAATGTAATTTAATTTCCATTTTCTTTTTAGAGCAGTATACAAAGATGCTGATTTGTATTTATTAGACTCTCCTTTTGGATACCTAGATGTTTTAACAGAAAAAGAAATATTTGAAAGGTATGTTCTTTGAATACCTTACTTATAATGCTCATGCTAAAATAAAAGAAAGACAGACTGTC**CCATCATAGATTGCATTTTACCTCTTGAGA**AATATGTTCACCATTGTTGGTATGGCAGAATGTAGCATGGTATTAACTCAAATCTGATCTGCCCTACTGGGCCAGGATTCAAGATTACTTCCATTAAAACCTTTTCTCACCGCCTCATG

**EXON 14**

Exon14_F1 5'-AGCATGTTATTTCATGCTATCAGAATTCAC-3'

Exon14_R1 5'-TGTTCCATTTTAGAAGTGACCAAAATCCT-3'

Exon14_F2 5'-TATCTTAAAGCTGTGTCTGTAAACTGATGG-3'

Exon14_R2 5'-CTTTCTGCACTAAATTGGTCGAAAGAAT-3'

Exon14_F3 5'-ACAGCCAGACTTTAGCTCAAAACT-3'

Exon14_R3 5'-ATCCTCTTCGATGCCATTCATTTGTA-3'

Exon14_F4 5'-CAACTCTATACGAAAATTTTCCATTGTGCA-3'

Exon14_R4 5'-CGGTGAATGTTCTGACCTTGGTTA-3'

Exon14_F5 5'-GCAGTCTGTCCTGAACCTGATG-3'

Exon14_R5 5'-ATTGCATTCTACTCAATTGCATTCTGTG-3'

PCR_exon14_F 5'-AAATAAACTGAGAGACCCCGAG-3'

PCR_exon14_R 5'-TTGCATTCTGTGGGGTGAAA-3'

Supplementary Figure 4 *(continued)*

AAATAAACTGAGAGACCCCGAGGATAAATGATTTGCTCAAAGTCAAATATCTACTTAATATAGGAAATTTAATTTCATTCTCAGTCTGTTAACATGCAACTTTTCAATAT**AGCATGTTATTTCATGCTATCAGAATTCAC**AAGGTACCAATTTAATTACTACAGAGTACTTATAGAATCATTTAAAATATAATAAAATTGTATGATAGAGATTATATGCAATAAAACATTAACAAAATGCTAAAATACGAGACATATTGCAATAAAGTATTTATAAAATTGATATTTATATGTTTTTA**TATCTTAAAGCTGTGTCTGTAAACTGATGG**CTAACAAAACT**AGGATTTTGGTCACTTCTAAAATGGAACA**TTTAAAGAAAGCTGACAAAATATTAATTTTGCATGAAGGTAGCAGCTATTTTTATGGGACATTTTCAGAACTCCAAAATCT**ACAGCCAGACTTTAGCTCAAAACT**CATGGGATGTG**ATTCTTTCGACCAATTTAGTGCAGAAAG**AAGAAATTCAATCCTAACTGAGACCTTACACCGTTTCTCATTAGAAGGAGATGCTCCTGTCTCCTGGACAGAAACAAAAAAACAATCTTTTAAACAGACTGGAGAGTTTGGGGAAAAAAGGAAGAATTCTATTCTCAATCCAAT**CAACTCTATACGAAAATTTTCCATTGTGCA**AAAGACTCCCT**TACAAATGAATGGCATCGAAGAGGAT**TCTGATGAGCCTTTAGAGAGAAGGCTGTCCTTAGTACCAGATTCTGAGCAGGGAGAGGCGATACTGCCTCGCATCAGCGTGATCAGCACTGGCCCCACGCTTCAGGCACGAAGGAG**GCAGTCTGTCCTGAACCTGATG**ACACACTCAGT**TAACCAAGGTCAGAACATTCACCG**AAAGACAACAGCATCCACACGAAAAGTGTCACTGGCCCCTCAGGCAAACTTGACTGAACTGGATATATATTCAAGAAGGTTATCTCAAGAAACTGGCTTGGAAATAAGTGAAGAAATTAACGAAGAAGACTTAAAGGTAGGTATACATCGCTTGGGGGTATTTCACCC**CACAGAATGCAATTGAGTAGAATGCAAT**

**EXON 15**

Exon15_F1 5'-ATTAAAAATAAAACCACAATGGTGGCATGA-3'

Exon15_R1 5'-AATACTTTACAATAGAACATTCTTACCTCTGC-3'

Exon15_F2 5'-ATTTGGTGCTTAGTAATTTTTCTGGCAG-3'

Exon15_R2 5'-ACTTGTTGATTTTTCAGAAGCTAAGAACTATAT-3'

PCR_exon15_F 5'-ACAATGGTGGCATGAAACTGT-3'

PCR_exon15_R 5'-GCCTTCTACTTTGAGCTTTCGA-3'

**ATTAAAAATAAAACCACAATGGTGGCATGA**AACTGTACTGTCTTATTGTAATAGCCATAATTCTTTTATTCAGGAGTGCTTTTTTGATGATATGGAGAGCATACCAGCAGTGACTACATGGAACACATACCTTCGATATATTACTGTCCACAAGAGCTTAATTTTTGTGCTA**ATTTGGTGCTTAGTAATTTTTCTGGCAGAGGTAAGAATGTTCTATTGTAAAGTATT**ACTGGATTTAAAGTTAAATTAAGATAGTTTGGGGATGTATACATATATATGCACACACATAAATATGTATATATACACATGTATACATGTATAAGTATGCATATATACACACATATATCACTATATGTATATATGTATATATTACATATATTTGTGATTTTACAGTATATAATGGTATAGATTC**ATATAGTTCTTAGCTTCTGAAAAATCAACAAGT**AGAACCACTACTGATATTTTATTATTTCATATTACATATAAAATATATTTAAATACAAATATAAGAAGAGTTTTTAATAGATTTTTAATAATAAAGGTTAAGAGATTCGAAAGCTCAAAGTAGAAGGC

**EXON 16**

Exon16_F 5'-GTGTACCTTGATATTGGTACACACATCA-3'

Exon16_R 5'-ATAATGCTTGGGAGAAATGAAACAAAGTG-3'

PCR_exon16_F 5'-GGCATGGGAGGAATAGGTGA-3'

PCR_exon16_R 5'-ACACTACAGCCCTGAACTCC-3'

**GTGTACCTTGATATTGGTACACACATCA**AATGGTGTGATGTGAATTTAGATGTGGGCATGGGAGGAATAGGTGAAGATGTTAGAAAAAAAATCAACTGTGTCTTGTTCCATTCCAGGTGGCTGCTTCTTTGGTTGTGCTGTGGCTCCTTGGAAAGTGAGTATTCCATGTCCTATTGTGTAGATTGTGTTTTATTTCTGTTGATTAAATATTGTAATCCACTATGTTTGTATGTATTGTAATC**CACTTTGTTTCATTTCTCCCAAGCATTAT**GGTAGTGGAAAGATAAGGTTTTTTGTTTAAATGATGACCATTAGTTGGGTGAGGTGACACATTCCTGTAGTCCTAGCTCCTCCACAGGCTGACGCAGGAGGATCACTTGAGCCCAGGAGTTCAGGGCTGTAGTGT

Supplementary Figure 4 *(continued)*

**EXON 17**

Exon17_F1 5'-GAGGTTAAGGGTGCATGCTCTT-3'

Exon17_R1 5'-CGGCTACTCCCACGTAAATGTAAAA-3'

Exon17_F2 5'-CAGTGATTATCACCAGCACCAGTT-3'

Exon17_R2 5'-GGATCAGCAGTTTCATTTCTTAGACCT-3'

PCR_exon17_F 5'-AGGTTAAGGGTGCATGCTCT-3'

PCR_exon17_R 5'-TGCTACAGTCCCACCAACAT-3'

**GAGGTTAAGGGTGCATGCTCTT**CTAATGCAAAATATTGTATTTATTTAGACTCAAGTTTAGTTCCATTTACATGTATTGGAAATTCAGTAAGTAACTTTGGCTGCCAAATAACGATTTCCTATTTGCTTTACAGCACTCCTCTTCAAGACAAAGGGAATAGTACTCATAGTAGAAATAACAGCTATG**CAGTGATTATCACCAGCACCAGTT**CGTATTATGTG**TTTTACATTTACGTGGGAGTAGCCG**ACACTTTGCTTGCTATGGGATTCTTCAGAGGTCTACCACTGGTGCATACTCTAATCACAGTGTCGAAAATTTTACACCACAAAATGTTACATTCTGTTCTTCAAGCACCTATGTCAACCCTCAACACGTTGAAAGCAGGTACTTTACT**AGGTCTAAGAAATGAAACTGCTGATCC**ACCATCAATAGGGCCTGTGGTTTTGTTGGTTTTCTAATGGCAGTGCTGGCTTTTGCACAGAGGCATGTGCCCTTTGTTGAACCTCCATTTGACTGGCATGCACATGTCTCAGATATTATAGGTTATCATATATTGTTGCTCCTAATATTTCTGTGTTAGATAATTAGAGTAGCTTGGTTTGTAAGAATGTGATGTTGGTGGGACTGTAGCA

**EXON 18**

Exon18_F1 5'-TTTGCTAATTCTTATTTGGGTTCTGAATGC-3'

Exon18_R1 5'-GTAAGAGGCAGAAGGTCATCCAA-3'

Exon18_F2 5'-AGGTGGGATTCTTAATAGATTCTCCAAAG-3'

Exon18_R2 5'-CAGGACTTCAACCCTCAATCAAATAAAAA-3'

PCR_exon18_F 5'-TGGGTTCTGAATGCGTCTACT-3'

PCR_exon18_R 5'-ATGGTCCTTTGTGCCTCTCA-3'

**TTTGCTAATTCTTATTTGGGTTCTGAATGC**GTCTACTGTGATCCAAACTTAGTATTGAATATATTGATATATCTTTAAAAAATTAGTGTTTTTTGAGGAATTTGTCATCTTGTATATTAT**AGGTGGGATTCTTAATAGATTCTCCAAAGA**TATAGCAATT**TTGGATGACCTTCTGCCTCTTAC**CATATTTGACTTCATCCAGGTATGTAAAAATAAGTACCGTTAAGTATGTCTGTATTATTAAAAAAACAATAACAAAAGCAAATGTGATTTTGTTTTCAT**TTTTTATTTGATTGAGGGTTGAAGTCCTG**TCTATTGCATTAATTTTGTAATTATCCAAAGCCTTCAAAATAGACATAAGTTTAGTAAATTCAATAATAAGTCAGAACTGCTTACCTGGCCCAAACCTGAGGCAATCCCACATTTAGATGTAATAGCTGTCTACTTGGGAGTGATTTGAGAGGCACAAAGGACCAT

**EXON 19**

Exon19_F1 5'-AGAAATAAATCACTGACACACTTTGTCCA-3'

Exon19_R1 5'-CTGTCATACCTTCAGATTCCAGTTGT-3'

Exon19_F2 5'-GAGAGCATATTTCCTCCAAACCTCA-3'

Exon19_R2 5'-AAAACCAAAATGAAGTCACATGGTCATTAA-3'

PCR_exon19_F 5'-ATGACGAGTTAGTGGGTGCA-3'

PCR_exon19_R 5'-CCAAAATGAAGTCACATGGTCA-3'

Supplementary Figure 4 *(continued)*

ATGACGAGTTAGTGGGTGCAGTGCACCAGCATGGCACATGTATACATATGTAACTAACCTGCACAATGTGCACATGTACCCTAAAACTTAAAGTATAATAAAAAAAATAAAAAAAAGTTTGAGGTGTTTAAAGTATGCAAAAAAAAAAAA**AGAAATAAATCACTGACACACTTTGTCCA**CTTTGCAATGTGAAAATGTTTACTCACCAACATGTTTTCTTTGATCTTACAGTTGTTATTAATTGTGATTGGAGCTATAGCAGTTGTCGCAGTTTTACAACCCTACATCTTTGTTGCAACAGTGCCAGTGATAGTGGCTTTTATTATGTT**GAGAGCATATTTCCTCCAAACCTCA**CAGCAACTCAA**ACAACTGGAATCTGAAGGTATGACAG**TGAATGTGCGATACTCATCTTGTAAAAAAGCTATAAGAGCTATTTGAGATTCTTTATTGTTAATCTACTTAAAAAAAATTCTGCTTTTAAACTTTTACATCATATAACAATAATTTTTTTCTACATGCATGTGTATATAAAAGGAAACTATATTACAAAGTACACATGGATTTTTTTTCTTAA**TTAATGACCATGTGACTTCATTTTGGTTTT**

**EXON 20**

Exon20_F1 5'-TCAAAGAATGGCACCAGTGTGA-3'

Exon20_R1 5'-GCTTTGTGGAACAGAGTTTCAAAGTAAG-3'

Exon20_F2 5'-GACTATGGACACTTCGTGCCTT-3'

Exon20_R2 5'-ACCTATAGAATGCAGCATTTTATTCATTGA-3'

PCR_exon20_F 5'-TCAAAGAATGGCACCAGTGT-3'

PCR_exon20_R 5'-CAATCTGTGTGCATCGGTTT-3'

**TCAAAGAATGGCACCAGTGTGA**AAAAAAGCTTTTTAACCAATGACATTTGTGATATGATTATTCTAATTTAGTCTTTTTCAGGTACAAGATATTATGAAATTACATTTTGTGTTTATGTTATTTGCAATGTTTTCTATGGAAATATTTCACAGGCAGGAGTCCAATTTTCACTCATCTTGTTACAAGCTTAAAAG**GACTATGGACACTTCGTGCCTT**CGGACGGCAGC**CTTACTTTGAAACTCTGTTCCACAAAGC**TCTGAATTTACATACTGCCAACTGGTTCTTGTACCTGTCAACACTGCGCTGGTTCCAAATGAGAATAGAAATGATTTTTGTCATCTTCTTCATTGCTGTTACCTTCATTTCCATTTTAACAACAGGTACTATGAACTCATTAACTTTAGCTAAGCATTTAAGTAAAAAATTT**TCAATGAATAAAATGCTGCATTCTATAGGT**TATCAATTTTTGATATCTTTAGAGTTTAGTAATTAACAAATTTGTTGGTTTATTATTGAACAAGTGATTTCTTTGAATTTCCATTGTTTTATTGTTAAACAAATAATTTCCTTGAAATCGGATATATATATATATATGTATATATATATATATATATATATATATATACATATATATATATAGTATTATCCCTGTTTTCACAGTTTTAAAAACCGATGCACACAGATTG

**EXON 21**

Exon21_F1 5'-GGGCAACACTTTCCTAATATTCAATCG-3'

Exon21_R1 5'-AGGATAATACCAACTCTTCCTTCTCCTT-3'

Exon21_F2 5'-ATGAGGTTCATTTACGTCTTTTGTGC-3'

Exon21_R2 5'-ACAGTGACCCTCAATTTATCTGTAATGTTT-3'

PCR_exon21_F 5'-TGTGCCCTAGGAGAAGTGTG-3'

PCR_exon21_R 5'-GGACAATTTGGCACCACTCA-3'

**GGGCAACACTTTCCTAATATTCAATCG**CTCTTTGATTTAAAATCCTGGTTGAATACTTACTATATGCAGAGCATTATTCTATTAGTAGATGCTGTGATGAACTGAGATTTAAAAATTGTTAAAATTAGCATAAAATTGAAATGTAAATTTAATGTGATATGTGCCCTAGGAGAAGTGTGAATAAAGTCGTTCACAGAAGAGAGAAATAAC**ATGAGGTTCATTTACGTCTTTTGTGC**ATCTATAGGAG**AAGGAGAAGGAAGAGTTGGTATTATCCT**GACTTTAGCCATGAATATCATGAGTACATTGCAGTGGGCTGTAAACTCCAGCATAGATGTGGATAGCTTGGTAAGTCTTATCATCTTTTTAACTTTTATGAAAAAAATTCAGACAAGTAACAAAGTATGAGTAATAGCATGAGGAAGAACTATATACCGTATATTGAGCTTAAGAAATA**AAACATTACAGATAAATTGAGGGTCACTGT**GTATCTGTCATTAAATCCTTATCTCTTCTTTCCTTCTCATAGATAGCCACTATGAAGATCTAATACTGCAGTGAGCATTCTTTCACCTGTTTCCTTATTCAGGATTTTCTAGGAGAAATACCTAGGGGTTGTATTGCTGGGTCATAGGATTCACCCATGCTTAACTGAGTGGTGCCAAATTGTCC

Supplementary Figure 4 *(continued)*

**EXON 22**

Exon22_F1 5'-AAGTCCTGGTTATTTCTCTTCAGTTAAACT-3'

Exon22_R1 5'-GGTTGACTTGGTAGGTTTACCTTCT-3'

Exon22_F2 5'-TGTGAGCCGAGTCTTTAAGTTCATT-3'

Exon22_R2 5'-CAGTCTAACAAAGCAAGCAGTGTT-3'

PCR_exon22_F 5'-GCCCGACAAATAACCAAGTGA-3'

PCR_exon22_R 5'-TCTGCTAACACATTGCTTCAGG-3'

**AAGTCCTGGTTATTTCTCTTCAGTTAAACT**TTTAATTATATCCAATTATTTCCTGTTAGTTCATTGAAAAGCCCGACAAATAACCAAGTGACAAATAGCAAGTGTTGCATTTTACAAGTTATTTTTTAGGAAGCATCAAACTAATTGTGAAATTGTCTGCCATTCTTAAAAACAAAAATGTTGTTATTTTTATTTCAGATGCGATC**TGTGAGCCGAGTCTTTAAGTTCATT**GACATGCCAAC**AGAAGGTAAACCTACCAAGTCAACC**AAACCATACAAGAATGGCCAACTCTCGAAAGTTATGATTATTGAGAATTCACACGTGAAGAAAGATGACATCTGGCCCTCAGGGGGCCAAATGACTGTCAAAGATCTCACAGCAAAATACACAGAAGGTGGAAATGCCATATTAGAGAACATTTCCTTCTCAATAAGTCCTGGCCAGAGGGTGAGATTTG**AACACTGCTTGCTTTGTTAGACTG**TGTTCAGTAAGTGAATCCCAGTAGCCTGAAGCAATGTGTTAGCAGA

**EXON 23**

Exon23_F1 5'-GAAAGTGTGCAACAAGGTTTGAATGA-3'

Exon23_R1 5'-AAGTTATTGAATCCCAAGACACACCAT-3'

Exon23_F2 5'-TTGAGACTACTGAACACTGAAGGAGA-3'

Exon23_R2 5'-CCTATGAGAAAACTGCACTGGAGAAAAA-3'

PCR_exon23_F 5'-CTGCATCAGGGGTCCAATTC-3'

PCR_exon23_R 5'-CCCAAGGCTCCCACTGTAAA-3'

CTGCATCAGGGGTCCAATTCCTTATGGCCAGTTTCTCTATTCTGTTCCAAGGTTGTTTGTCTCCATATATCAACATTGGTCAGGATT**GAAAGTGTGCAACAAGGTTTGAATGA**ATAAGTGAAAATCTTCCACTGGTGACAGGATAAAATATTCCAATGGTTTTTATTGAAGTACAATACTGAATTATGTTTATGGCATGGTACCTATATGTCACAGAAGTGATCCCATCACTTTTACCTTATAGGTGGGCCTCTTGGGAAGAACTGGATCAGGGAAGAGTACTTTGTTATCAGCTTTT**TTGAGACTACTGAACACTGAAGGAGA**AATCCAGATCG**ATGGTGTGTCTTGGGATTCAATAACTT**TGCAACAGTGGAGGAAAGCCTTTGGAGTGATACCACAGGTGAGCAAAAGGACTTAGCCAGAAAAAAGGCAACTAAATTATATTTTTTACTGCTATTTGATACTTGTACTCAAGAAATTCATATTACTCTGCAAAATATATTTGTTATGCATTGCTGTCT**TTTTTCTCCAGTGCAGTTTTCTCATAGG**CAGAAAAGATGTCTCTAAAAGTTTGGAATTCTCAAATTCTGGTTATTGAAATGTTCATAGCTTTGATAGTGTTTTTCAGAAGACCAAATTTACAGTGGGAGCCTTGGG

**EXON 24**

Exon24_F1 5'-TTTGAGAGAACTTGATGGTAAGTACATGG-3'

Exon24_R1 5'-CCTTACCTCATCTGCAACTTTCCATAT-3'

Exon24_F2 5'-ACTTGGATCCCTATGAACAGTGGA-3'

Exon24_R2 5'-GTCAGCTATATCAGCCATTTGTGTTG-3'

PCR_exon24_F 5'-GGGACTCCAAATATTGCTGTAGT-3'

PCR_exon24_R 5'-ACCAAGATAGGGCCTGATGG-3'

Supplementary Figure 4 *(continued)*

GGGACTCCAAATATTGCTGTAGTATTTGTTTCTTAAAAGAATGATACAAAGCAGACATGATAAAATATTAAAA**TTTGAGAGAACTTGATGGTAAGTACATGG**GTGTTTCTTATTTTAAAATAATTTTTCTACTTGAAATATTTTACAATACAATAAGGGAAAAATAAAAAGTTATTTAAGTTATTCATACTTTCTTCTTCTTTTCTTTTTTGCTATAGAAAGTATTTATTTTTTCTGGAACATTTAGAAAAA**ACTTGGATCCCTATGAACAGTGGA**GTGATCAAGAA**ATATGGAAAGTTGCAGATGAGGTAAGG**CTGCTAACTGAAATGATTTTGAAAGGGGTAACTCATAC**CAACACAAATGGCTGATATAGCTGAC**ATCATTCTACACACTTTGTGTGCATGTATGTGTGTGCACAACTTTAAAATGGAGTACCCTAACATACCTGGAGCAACAGGTACTTTTGACTGGACCTACCCCTAACTGAAATGATTTTGAAAGAGGTAACTCATACCAACACAAATGGTTGATATGGCTAAGATCATTCTACACACTTTGTGTGCATGTATTTCTGTGCACAACTTCAAAATGGAGTACCCTAAAATACCTGGCGCGACAAGTACTTTTGACTGAGCCTACTTCTCTCCTCACTGGTATGGCTCCAACCATCAGGCCCTATCTTGGT

**EXON 25**

Exon25_F 5'-GCTTGAGTGTTTTTAACTCTGTGGTATC-3'

Exon25_R 5'-AATGATTCTGTTCCCACTGTGCTATTAA-3'

PCR_exon25_F 5'-CAAATGGTGGCAGGTAGTGG-3'

PCR_exon25_R 5'-ACGCAGACATGACAGCCTAA-3'

CAAATGGTGGCAGGTAGTGGGGGTAGAGGGATTGGTATGAAAAACATAAGCTTTCAGAACTCCTGTGTTTATTTTTAGAATGTCAACT**GCTTGAGTGTTTTTAACTCTGTGGTATC**TGAACTATCTTCTCTAACTGCAGGTTGGGCTCAGATCTGTGATAGAACAGTTTCCTGGGAAGCTTGACTTTGTCCTTGTGGATGGGGGCTGTGTCCTAAGCCATGGCCACAAGCAGTTGATGTGCTTGGCTAGATCTGTTCTCAGTAAGGCGAAGATCTTGCTGCTTGATGAACCCAGTGCTCATTTGGATCCAGTGTGAGTTTCAGATGTTCTGTTAC**TTAATAGCACAGTGGGAACAGAATCATT**ATGCCTGCTTCATGGTGACACATATTTCTATTAGGCTGTCATGTCTGCGT

**EXON 26**

Exon26_F1 5'-CCCATGGTTGAAAAGCTGATTGT-3'

Exon26_R1 5'-GCTTCTATCCTGTGTTCACAGAGAA-3'

Exon26_F2 5'-GAAGAACTCTAAAACAAGCATTTGCTGA-3'

Exon26_R2 5'-AAGAATTACAAGGGCAATGAGATCTTAAGT-3'

PCR_exon26_F 5'-GCCCCAAATAAAGAAGTACTGGT-3'

PCR_exon26_R 5'-CCACATGGCTCAGATCAAAGT-3'

GCCCCAAATAAAGAAGTACTGGTGATTCTACATAATGAAAAATGTACTCATTTATTAAAGTTTCTTTGAAATATTTGTCCTGTTTATTTATGGATACTTAGAGTCTAC**CCCATGGTTGAAAAGCTGATTGT**GGCTAACGCTATATCAACATTATGTGAAAAGAACTTAAAGAAATAAGTAATTTAAAGAGATAATAGAACAATAGACATATTATCAAGGTAAATACAGATCATTACTGTTCTGTGATATTATGTGTGGTATTTTCTTTCTTTTCTAGAACATACCAAATAATTA**GAAGAACTCTAAAACAAGCATTTGCTGA**TTGCACAGTAA**TTCTCTGTGAACACAGGATAGAAGC**AATGCTGGAATGCCAACAATTTTTGGTGAGTCTTTATAACTTT**ACTTAAGATCTCATTGCCCTTGTAATTCTT**GATAACAATCTCACATGTGATAGTTCCTGCAAATTGCAACAATGTACAAGTTCTTTTCAAAAATATGTATCATACAGCCATCCAGCTTTACTCAAAATAGCTGCACAAGTTTTTCACTTTGATCTGAGCCATGTGG

Supplementary Figure 4 *(continued)*

**EXON 27**

Exon27_F 5'-CCTTCTGTCCCAGATCTCACTAAC-3'

Exon27_R 5'-TGAGCAAATGTCCCATGTCAACA-3'

PCR_exon27_F 5'-AGTTTCTGTCCCTGCTCTGG-3'

PCR_exon27_R 5'-TCTGTGGAAAGAAGGGCTGT-3'

AGTTTCTGTCCCTGCTCTGGTCTGACCTG**CCTTCTGTCCCAGATCTCACTAAC**AGCCATTTCCCTAGGTCATAGAAGAGAACAAAGTGCGGCAGTACGATTCCATCCAGAAACTGCTGAACGAGAGGAGCCTCTTCCGGCAAGCCATCAGCCCCTCCGACAGGGTGAAGCTCTTTCCCCACCGGAACTCAAGCAAGTGCAAGTCTAAGCCCCAGATTGCTGCTCTGAAAGAGGAGACAGAAGAAGAGGTGCAAGATACAAGGCTTTAGAGAGCAGCATAAA**TGTTGACATGGGACATTTGCTCA**TGGAATTGGAGCTCGTGGGACAGTCACCTCATGGAATTGGAGCTCGTGGAACAGTTACCTCTGCCTCAGAAAACAAGGATGAATTAAGTTTTTTTTTAAAAAAGAAACATTTGGTAAGGGGAATTGAGGACACTGATATGGGTCTTGATAAATGGCTTCCTGGCAATAGTCAAATTGTGTGAAAGGTACTTCAAATCCTTGAAGATTTACCACTTGTGTTTTGCAAGCCAGATTTTCCTGAAAACCCTTGCCATGTGCTAGTAATTGGAAAGGCAGCTCTAAATGTCAATCAGCCTAGTTGATCAGCTTATTGTCTAGTGAAACTCGTTAATTTGTAGTGTTGGAGAAGAACTGAAATCATACTTCTTAGGGTTATGATTAAGTAATGATAACTGGAAACTTCAGCGGTTTATATAAGCTTGTATTCCTTTTTCTCTCCTCTCCCCATGATGTTTAGAAACACAACTATATTGTTTGCTAAGCATTCCAACTATCTCATTTCCAAGCAAGTATTAGAATACCACAGGAACCACAAGACTGCACATCAAAATATGCCCCATTCAACATCTAGTGAGCAGTCAGGAAAGAGAACTTCCAGATCCTGGAAATCAGGGTTAGTATTGTCCAGGTCTACCAAAAATCTCAATATTTCAGATAATCACAATACATCCCTTACCTGGGAAAGGGCTGTTATAATCTTTCACAGGGGACAGGATGGTTCCCTTGATGAAGAAGTTGATATGCCTTTTCCCAACTCCAGAAAGTGACAAGCTCACAGACCTTTGAACTAGAGTTTAGCTGGAAAAGTATGTTAGTGCAAATTGTCACAGGACAGCCCTTCTTTCCACAGA

Supplementary Figure 4 *(continued)*


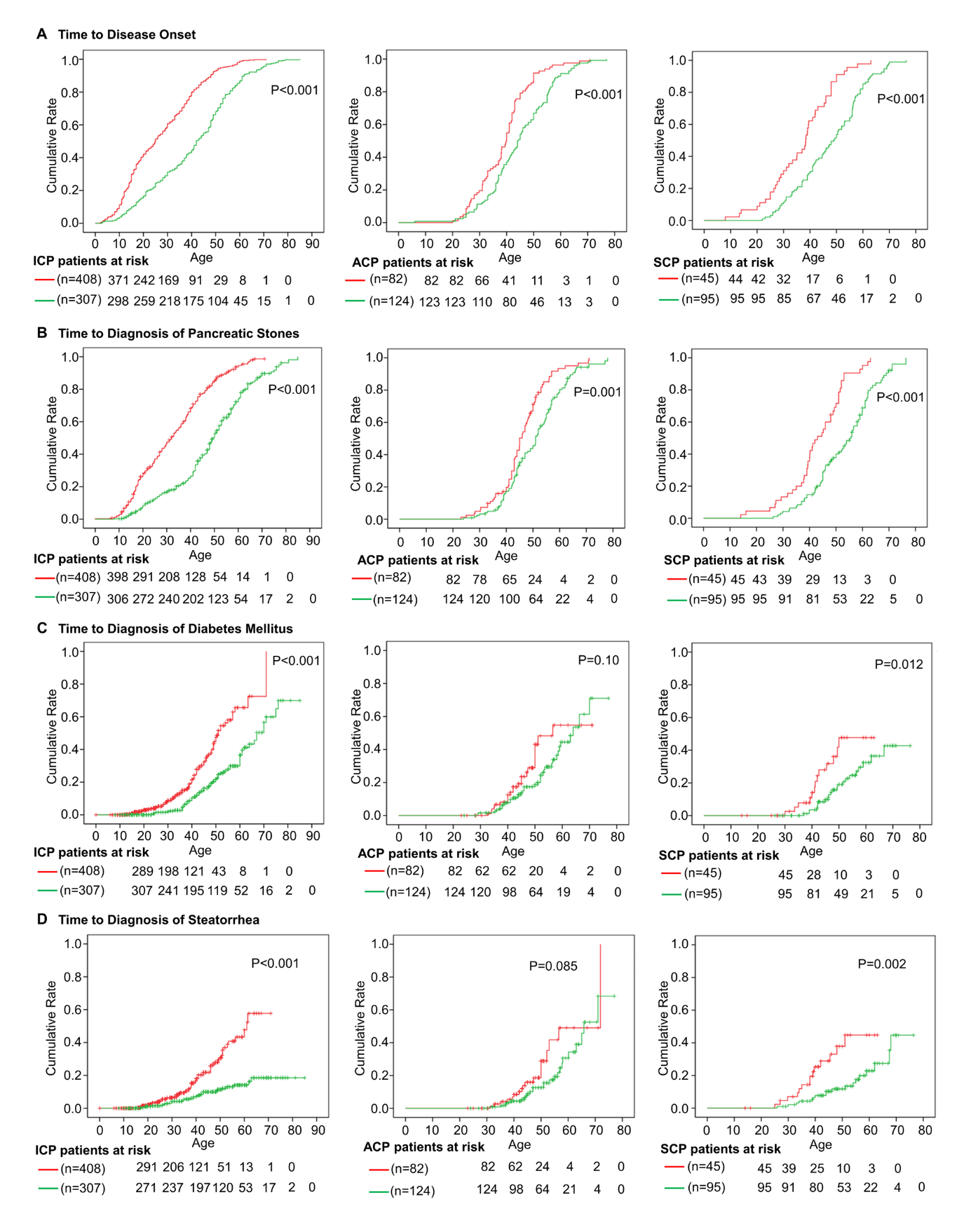


**Supplementary Figure 5.** Pathogenic genotypes affect disease onset and clinical outcomes of ICP, ACP and SCP. Kaplan-Meier plots of age at disease onset (**A**), age at diagnosis of pancreatic stones (**B**), age at diagnosis of diabetes mellitus (**C**) and age at diagnosis of steatorrhea (**D**) for Han Chinese ICP, ACP and SCP patients with and without pathogenic *SPINK1*, *PRSS1*, *CTRC* and/or *CFTR* genotypes. Red, patients with pathogenic genotypes. Blue, patients without pathogenic genotypes.

**Supplementary Table 1.** Carrier Frequencies of Rare Pathogenic *SPINK1, PRSS1*, *CTRC* and *CFTR* Variants in all Han Chinese CP Patients and Controls

| Variant | | | Patients  (n = 1061) | | Controls  (n = 1196) | | OR | 95% CI | *P* value | Variant status^*^ |
| --- | --- | --- | --- | --- | --- | --- | --- | --- | --- | --- |
| Gene | Nucleotide  change† | Amino acid change | + | % | + | % |  |  |  |  |
| *SPINK1* | c.88-1G>A |  | 1 | 0.09 | 0 | 0 |  |  | NS# | Novel |
|  | c.93_101del | p.K31_Y33del | 4 | 0.38 | 0 | 0 |  |  | NS | Previously reported |
|  | c.101A>G | p.N34S | 20 | 1.89 | 5 | 0.42 | 4.58 | 1.71-12.24 | <0.001 | Previously reported |
|  | c.101A>G (hom) | p.N34S (hom) | 1 | 0.09 | 0 | 0 |  |  | NS | Previously reported |
|  | c.142G>A | p.G48R | 2 | 0.19 | 0 | 0 |  |  | NS | Novel |
|  | c.172T>A | p.C58S | 1 | 0.09 | 0 | 0 |  |  | NS | Novel |
|  | c.174C>A | p.C58X | 0 | 0.00 | 1 | 0.08 |  |  | NS | Novel |
|  | c.194G>A | p.R65Q | 1 | 0.09 | 0 | 0 |  |  | NS | Previously reported |
|  | c.194+2T>C |  | 328 | 30.91 | 13 | 1.09 | 40.72 | 23.21-71.43 | <0.001 | Previously reported |
|  | c.194+2T>C (hom) |  | 57 | 5.37 | 0 | 0 | 137 | 8.45-2221 | <0.001 | Previously reported |
|  | c.199C>T | p.R67C | 1 | 0.09 | 0 | 0 |  |  | NS | Previously reported |
|  | c.202C>T | p.Q68X | 1 | 0.09 | 0 | 0 |  |  | NS | Novel |
|  | c.206C>T | p.T69I | 3 | 0.28 | 0 | 0 |  |  | NS | Previously reported |
| Total | | | 420 | 39.59 | 19 | 1.59 | 40.59 | 25.37-64.93 | <0.001 |  |
| *PRSS1* | c.86A>T | p.N29I | 8 | 0.75 | 0 | 0 | 19.31 | 1.11-335.1 | 0.008 | Previously reported |
|  | c.346C>T | p.R116C | 14 | 1.32 | 0 | 0 | 33.13 | 1.97-556.4 | <0.001 | Previously reported |
|  | c.364C>T | p.R122C | 3 | 0.28 | 0 | 0 |  |  | NS | Previously reported |
|  | c.365G>A | p.R122H | 22 | 2.07 | 3 | 0.25 | 8.42 | 2.51-28.22 | <0.001 | Previously reported |
|  | c.544A>T | p.N182Y | 1 | 0.09 | 0 | 0 |  |  | NS | Novel |
|  | c.623G>C | p.G208A | 87 | 8.20 | 22 | 1.84 | 4.77 | 2.96-7.67 | <0.001 | Previously reported |
|  | c.623G>C (hom) | p.G208A (hom) | 2 | 0.19 | 0 | 0 |  |  | NS | Previously reported |
| Total | | | 137 | 12.91 | 25 | 2.09 | 6.95 | 4.50-10.73 | <0.001 | Previously reported |
| *CTRC* | c.2T>C | p.M1T | 0 | 0.00 | 1 | 0.08 |  |  | NS | Novel |
|  | c.86G>C | p.R29P | 2 | 0.19 | 0 | 0 |  |  | NS | Novel |
|  | c.94G>A | p.G32R | 2 | 0.19 | 0 | 0 |  |  | NS | Novel |
|  | c.176G>A | p.C59Y | 0 | 0 | 1 | 0.08 |  |  | NS | Novel |
|  | c.180C>T | p.G60G | 18 | 1.70 | 3 | 0.25 | 6.86 | 2.02-23.37 | <0.001 | Previously reported |
|  | c.181G>A | p.G61R | 1 | 0.09 | 0 | 0 |  |  | NS | Previously reported |
|  | c.217G>T | p.A73S | 1 | 0.09 | 1 | 0.08 |  |  | NS | Novel |
|  | c.493+1G>A |  | 1 | 0.09 | 0 | 0 |  |  | NS | Novel |
|  | c.641G>A | p.G214E | 1 | 0.09 | 0 | 0 |  |  | NS | Novel |
|  | c.649G>A | p.G217S | 1 | 0.09 | 0 | 0 |  |  | NS | Previously reported |
|  | c.703G>A | p.V235I | 1 | 0.09 | 0 | 0 |  |  | NS | Previously reported |
|  | c.760C>T | p.R254W | 1 | 0.09 | 2 | 0.17 |  |  | NS | Previously reported |
| Total | | | 25 | 2.36 | 8 | 0.67 | 3.58 | 1.61-7.98 | <0.001 |  |
| *CFTR* | c. 263T>G | p.L88X | 0 | 0 | 1 | 0.08 |  |  | NS | Previously reported |
|  | c.1351G>A | p.G451R | 1 | 0.09 | 0 | 0 |  |  | NS | Novel |
|  | c.1390A>C | p.K464Q | 1 | 0.09 | 0 | 0 |  |  | NS | Novel |
|  | c.1488G>T | p.W496C | 2 | 0.19 | 0 | 0 |  |  | NS | Novel |
|  | c.1549T>C | p.Y517H | 0 | 0 | 1 | 0.08 |  |  | NS | Novel |
|  | c.1630G>T | p.G544C | 1 | 0.09 | 0 | 0 |  |  | NS | Novel |
|  | c.1813T>C | p.S605P | 0 | 0 | 1 | 0.08 |  |  | NS | Novel |
|  | c.1858C>T | p.H620Y | 0 | 0 | 1 | 0.08 |  |  | NS | Novel |
|  | c.1865G>A | p.G622D | 1 | 0.09 | 1 | 0.08 |  |  | NS | Previously reported |
|  | c.2173G>A | p.E725K | 1 | 0.09 | 0 | 0 |  |  | NS | Previously reported |
|  | c.2909G>A | p.G970D | 8 | 0.75 | 1 | 0.08 |  |  | NS | Previously reported |
|  | c.2936A>C | p.D979A | 3 | 0.28 | 0 | 0 |  |  | NS | Previously reported |
|  | c.3205G>A | p.G1069R | 10 | 0.94 | 1 | 0.08 | 11.37 | 1.45-89.01 | 0.004 | Previously reported |
|  | c.3635delT | p.V1212fs | 1 | 0.09 | 0 | 0 |  |  | NS | Novel |
|  | c.3987_3988del | p.E1329fs | 0 | 0 | 1 | 0.08 |  |  | NS | Novel |
|  | c.4056G>C | p.Q1352H | 31 | 2.92 | 11 | 0.92 | 3.24 | 1.62-6.48 | <0.001 | Previously reported |
| Total | | | 60 | 5.66 | 19 | 1.59 | 3.71 | 2.20-6.26 | <0.001 |  |

^*^Variants were divided into previously reported or novel categories by reference to data in the Genetic Risk Factors in Chronic Pancreatitis Database (http://www.pancreasgenetics.org/index.php) (for *SPINK1*, *PRSS1* and *CTRC* variants) or *CFTR2* (https://www.cftr2.org/) and *CFTR*-France (https://cftr.iurc.montp.inserm.fr/cgi-bin/about_CFTR.cgi) databases (for *CFTR* variants) (as of May 31, 2018).

†Heterozygous state unless otherwise stated. Hom, homozygote.

^#^NS, not significant.

**Supplementary Table 2.** Rare Pathogenic Genotypes Involving the *SPINK1*, *PRSS1*, *CTRC* and/or *CFTR* Genes in all Han Chinese CP Patients and Controls

| Gene(s) | Genotype^*^ | Patients  (n = 1061) | | Controls  (n = 1196) | | OR | 95% CI | *P* Value |
| --- | --- | --- | --- | --- | --- | --- | --- | --- |
|  |  | **+** | **%** | **+** | **%** |  |  |  |
| *SPINK1* only | c.[88-1G>A];[=] | 1 | 0.09 | 0 | 0 |  |  | NS† |
|  | c.[93_101del];[=] | 3 | 0.28 | 0 | 0 |  |  | NS |
|  | c.[101A>G];[=] | 9 | 0.85 | 5 | 0.42 |  |  | NS |
|  | c.[142G>A];[=] | 2 | 0.19 | 0 | 0 |  |  | NS |
|  | c.[174C>A];[=] | 0 | 0 | 1 | 0.08 |  |  | NS |
|  | c.[194G>A];[=] | 1 | 0.09 | 0 | 0 |  |  | NS |
|  | c.[194+2T>C];[=] | 239 | 22.53 | 13 | 1.09 | 26.46 | 15.03-46.57 | <0.001 |
|  | c.[202C>T];[=] | 1 | 0.09 | 0 | 0 |  |  | NS |
|  | c.[101A>G];[101A>G] | 1 | 0.09 | 0 | 0 |  |  | NS |
|  | c.[194+2T>C];[194+2T>C] | 54 | 5.09 | 0 | 0 | 129.40 | 7.98-2100 | <0.001 |
|  | c.194+2T>C(;)93_101del | 1 | 0.09 | 0 | 0 |  |  | NS |
|  | c.194+2T>C(;)101A>G | 10 | 0.94 | 0 | 0 | 23.90 | 1.40-408.6 | 0.002 |
|  | c.194+2T>C(;)172T>A | 1 | 0.09 | 0 | 0 |  |  | NS |
|  | c.194+2T>C(;)206C>T | 3 | 0.28 | 0 | 0 |  |  | NS |
|  | Subtotal | 326 | 30.73 | 19 | 1.59 | 27.48 | 17.14-44.04 | <0.001 |
| *PRSS1* only | c.[86A>T];[=] | 7 | 0.66 | 0 | 0 | 17.02 | 0.97-298.6 | 0.015 |
|  | c.[346C>T];[=] | 12 | 1.13 | 0 | 0 | 28.50 | 1.68-482.3 | <0.001 |
|  | c.[364C>T];[=] | 3 | 0.28 | 0 | 0 |  |  | NS |
|  | c.[365G>A];[=] | 19 | 1.79 | 3 | 0.25 | 7.25 | 2.14-24.58 | <0.001 |
|  | c.[544A>T];[=] | 1 | 0.09 | 0 | 0 |  |  | NS |
|  | c.[623G>C];[=] | 39 | 3.68 | 22 | 1.84 | 2.04 | 1.20-3.46 | 0.007 |
|  | c.346C>T(;)623G>C | 1 | 0.09 | 0 | 0 |  |  | NS |
|  | c.[623G>C];[623G>C] | 1 | 0.09 | 0 | 0 |  |  | NS |
|  | Subtotal | 83 | 7.82 | 25 | 2.09 | 3.98 | 2.52-6.27 | <0.001 |
| *CTRC* only | c.[2T>C];[=] | 0 | 0 | 1 | 0.08 |  |  | NS |
|  | c.[86G>C];[=] | 2 | 0.19 | 0 | 0 |  |  | NS |
|  | c.[94G>A];[=] | 1 | 0.09 | 0 | 0 |  |  | NS |
|  | c.[176G>A];[=] | 0 | 0 | 1 | 0.08 |  |  | NS |
|  | c.[180C>T];[=] | 5 | 0.47 | 3 | 0.25 |  |  | NS |
|  | c.[217G>T];[=] | 1 | 0 | 1 | 0.08 |  |  | NS |
|  | c.[760C>T];[=] | 1 | 0 | 2 | 0.17 |  |  | NS |
|  | c.649G>A(;)703G>A | 1 | 0.09 | 0 | 0 |  |  | NS |
|  | Subtotal | 11 | 1.04 | 8 | 0.67 |  |  | NS |
| *CFTR* only | c.[263T>G];[=] | 0 | 0 | 1 | 0.08 |  |  | NS |
|  | c.[1390A>C];[=] | 1 | 0.09 | 0 | 0 |  |  | NS |
|  | c.[1488G>T];[=] | 2 | 0.19 | 0 | 0 |  |  | NS |
|  | c.[1549T>C];[=] | 0 | 0 | 1 | 0.08 |  |  | NS |
|  | c.[1630G>T];[=] | 1 | 0.09 | 0 | 0 |  |  | NS |
|  | c.[1813T>C];[=] | 0 | 0 | 1 | 0.08 |  |  | NS |
|  | c.[1858C>T];[=] | 0 | 0 | 1 | 0.08 |  |  | NS |
|  | c.[1865G>A];[=] | 1 | 0.09 | 1 | 0.08 |  |  | NS |
|  | c.[2909G>A];[=] | 5 | 0.47 | 1 | 0.08 |  |  | NS |
|  | c.[3205G>A];[=] | 3 | 0.28 | 1 | 0.08 |  |  | NS |
|  | c.[3635delT];[=] | 1 | 0.09 | 0 | 0 |  |  | NS |
|  | c.[3987_3988del];[=] | 0 | 0 | 1 | 0.08 |  |  | NS |
|  | c.[4056G>C];[=] | 11 | 1.04 | 11 | 0.92 |  |  | NS |
|  | Subtotal | 25 | 2.36 | 19 | 1.59 |  |  | NS |
| *SPINK1* and *PRSS1* | *SPINK1*:c.[194+2T>C] *PRSS1*:c.[86A>T] | 1 | 0.09 | 0 | 0 |  |  | NS |
|  | *SPINK1*:c.[194+2T>C];[194+2T>C] *PRSS1*:c.[365G>A] | 1 | 0.09 | 0 | 0 |  |  | NS |
|  | *SPINK1*:c.[194+2T>C];[194+2T>C] *PRSS1*:c.[623G>C] | 1 | 0.09 | 0 | 0 |  |  | NS |
|  | *SPINK1*:c.[194+2T>C] *PRSS1*:c.[623G>C];[623G>C] | 1 | 0.09 | 0 | 0 |  |  | NS |
|  | *SPINK1*:c.[194+2T>C] *PRSS1*:c.[623G>C] | 37 | 3.49 | 0 | 0 | 87.59 | 5.37-1429 | <0.001 |
|  | Subtotal | 41 | 3.86 | 0 | 0 | 97.31 | 5.98-1585 | <0.001 |
| *SPINK1* and *CTRC* | *SPINK1*:c.[194+2T>C] *CTRC*:c.[180C>T] | 8 | 0.75 | 0 | 0 | 19.31 | 1.11-335.1 | 0.008 |
|  | *SPINK1*:c.[194+2T>C] *CTRC*:c.[181G>A] | 1 | 0.09 | 0 | 0 |  |  | NS |
|  | *SPINK1*:c.[194+2T>C] *CTRC*:c.[493+1G>A] | 1 | 0.09 | 0 | 0 |  |  | NS |
|  | *SPINK1*:c.[194+2T>C] *CTRC*:c.[641G>A] | 1 | 0.09 | 0 | 0 |  |  | NS |
|  | Subtotal | 11 | 1.04 | 0 | 0 | 26.20 | 1.54-445.4 | <0.001 |
| *PRSS1* and *CTRC* | *PRSS1*:c.[365G>A] *CTRC*:c.[94G>A] | 1 | 0.09 | 0 | 0 |  |  | NS |
|  | *PRSS1*:c.[365G>A] *CTRC*:c.[180C>T] | 1 | 0.09 | 0 | 0 |  |  | NS |
| *SPINK1* and *CFTR* | *SPINK1*:c.[101A>G] *CFTR*:c.[2909G>A] | 1 | 0.09 | 0 | 0 |  |  | NS |
|  | *SPINK1*:c.[194+2T>C] *CFTR*:c.[1351G>A] | 1 | 0.09 | 0 | 0 |  |  | NS |
|  | *SPINK1*:c.[194+2T>C] *CFTR*:c.[2909G>A] | 2 | 0.19 | 0 | 0 |  |  | NS |
|  | *SPINK1*:c.[194+2T>C] *CFTR*:c.[2936A>C] | 2 | 0.19 | 0 | 0 |  |  | NS |
|  | *SPINK1*:c.[194+2T>C] *CFTR*:c.[3205G>A] | 2 | 0.19 | 0 | 0 |  |  | NS |
|  | *SPINK1*:c.[194+2T>C] *CFTR*:c.[4056G>C] | 13 | 1.23 | 0 | 0 | 30.81 | 1.83-519.3 | <0.001 |
|  | *SPINK1*:c.[194+2T>C];[194+2T>C] *CFTR*:c.[4056G>C] | 1 | 0.09 | 0 | 0 |  |  | NS |
|  | *SPINK1*:c.194+2T>C(;)199C>T *CFTR*:c.[2173G>A] | 1 | 0.09 | 0 | 0 |  |  | NS |
|  | Subtotal | 23 | 2.17 | 0 | 0 | 54.15 | 3.28-893.2 | <0.001 |
| *PRSS1* and *CFTR* | *PRSS1*:c.[346C>T] *CFTR*:c.[4056G>C] | 1 | 0.09 | 0 | 0 |  |  | NS |
|  | *PRSS1*:c.[623G>C] *CFTR*:c.[2936A>C] | 1 | 0.09 | 0 | 0 |  |  | NS |
|  | *PRSS1*:c.[623G>C] *CFTR*:c.[3205G>A] | 3 | 0.28 | 0 | 0 |  |  | NS |
|  | *PRSS1*:c.[623G>C] *CFTR*:c.[4056G>C] | 2 | 0.19 | 0 | 0 |  |  | NS |
|  | Subtotal | 7 | 0.66 | 0 | 0 | 17.02 | 0.97-298.6 | 0.015 |
| *CTRC and CFTR* | *CTRC*:c.[180C>T] *CFTR*:c.[3205G>A] | 2 | 0.19 | 0 | 0 |  |  | NS |
|  | *CTRC*:c.[180C>T] *CFTR*:c.[4056G>C] | 1 | 0.09 | 0 | 0 |  |  | NS |
|  | Subtotal | 3 | 0.28 | 0 | 0 |  |  | NS |
| *SPINK1, PRSS1* and *CTRC* | *SPINK1*:c.[194+2T>C] *PRSS1*:c.[623G>C] *CTRC*:c.[180C>T] | 1 | 0.09 | 0 | 0 |  |  | NS |
| *SPINK1, PRSS1* and *CFTR* | *SPINK1*:c.[194+2T>C] *PRSS1*:c.[623G>C] *CFTR*:c.[4056G>C] | 2 | 0.19 | 0 | 0 |  |  | NS |
|  | Total | 535 | 50.42 | 71 | 5.94 | 16.12 | 12.32-21.08 | <0.001 |

^*^Nomenclature following HGVS recommendations (http://varnomen.hgvs.org/).

^†^NS, not significant.

**Supplementary Table 3.** Rare Pathogenic Genotypes Involving the *SPINK1*, *PRSS1*, *CTRC* and/or *CFTR* Genes in Han Chinese ACP and SCP Patients

| Gene(s) | Genotype* | ACP (n = 206) | | SCP (n = 140) | |
| --- | --- | --- | --- | --- | --- |
|  |  | + | % | + | % |
| *SPINK1* only | c.[88-1G>A];[=] | 1 | 0.49 | 0 | 0 |
|  | c.[93_101del];[=] | 1 | 0.49 | 0 | 0 |
|  | c.[101A>G];[=] | 1 | 0.49 | 1 | 0.71 |
|  | c.[142G>A];[=] | 0 | 0 | 1 | 0.71 |
|  | c.[194+2T>C];[=] | 39 | 18.93 | 21 | 15.0 |
|  | c.[194+2T>C] ;[194+2T>C] | 6 | 2.91 | 3 | 2.14 |
|  | c.194+2T>C(;)101A>G | 1 | 0.49 | 0 | 0 |
|  | Subtotal | 49 | 23.79 | 26 | 18.57 |
| *PRSS1* only | c.[346C>T];[=] | 5 | 2.43 | 0 | 0 |
|  | c.[365G>A];[=] | 2 | 0.97 | 1 | 0.71 |
|  | c.[623G>C];[=] | 11 | 5.34 | 9 | 6.43 |
|  | c.[623G>C];[623G>C] | 1 | 0.49 | 0 | 0 |
|  | Subtotal | 19 | 9.22 | 10 | 7.14 |
| *CTRC* only | c.[86G>C];[=] | 0 | 0 | 2 | 1.43 |
|  | c.[180C>T];[=] | 1 | 0.49 | 0 | 0 |
|  | c.[217G>T];[=] | 0 | 0 | 1 | 0.71 |
|  | c.[760C>T];[=] | 1 | 0.49 | 0 | 0 |
|  | Subtotal | 2 | 0.97 | 3 | 2.14 |
| *CFTR* only | c.[1390A>C];[=] | 0 | 0 | 1 | 0.71 |
|  | c.[2909G>A];[=] | 1 | 0.49 | 0 | 0 |
|  | c.[4056G>C];[=] | 3 | 1.46 | 0 | 0 |
|  | Subtotal | 4 | 1.94 | 1 | 0.71 |
| *SPINK1* and *PRSS1* | *SPINK1*:c.[194+2T>C] *PRSS1*:c.[623G>C] | 3 | 1.46 | 3 | 2.14 |
|  | *SPINK1*:c.[194+2T>C];[194+2T>C] *PRSS1*:c.[623G>C] | 0 | 0 | 1 | 0.71 |
| *SPINK1* and *CFTR* | *SPINK1*:c.[194+2T>C] *CFTR*:c.[1351G>A] | 1 | 0.49 | 0 | 0 |
|  | *SPINK1*:c.[194+2T>C] *CFTR*:c.[4056G>C] | 2 | 0.97 | 1 | 0.71 |
| *PRSS1* and *CFTR* | *PRSS1*:c.[623G>C] *CFTR*:c.[4056G>C] | 1 | 0.49 | 0 | 0 |
| *SPINK1*, *PRSS1* and *CTRC* | *SPINK1*:c.[194+2T>C] *PRSS1*:c.[623G>C] *CTRC*:c.[180C>T] | 1 | 0.49 | 0 | 0 |
| Total | | 82 | 39.81 | 45 | 32.14 |

^*^Nomenclature following HGVS recommendations (http://varnomen.hgvs.org/).

**Supplementary Table 4.** Comparison of the Impact of Rare Pathogenic Genotypes Involving *SPINK1*, *PRSS1*, *CTRC* and/or *CFTR* Genes on Onset and Clinical Outcomes of Chronic Pancreatitis

| Clinical parameter | Mutation-positive patients (A) | Mutation-negative patients (B) | Median age difference between A and B | *P* value |
| --- | --- | --- | --- | --- |
| Median age at disease onset (years (95% CI))  All patients  ICP  ACP  SCP | 29.8 (27.7-31.9)  25.0 (22.8-27.8)  39.8 (37.5-42.1)  38.4 (35.8-41.0) | 44.2 (42.4-46.0)  42.0 (39.6-44.4)  44.0 (41.7-46.3)  49.0 (45.1-52.9) | 14.4  17  4.2  10.6 | < 0.001  < 0.001  < 0.001  < 0.001 |
|  | | | | |
| Median age at diagnosis of pancreatic stones (years (95% CI))  All patients  ICP  ACP  SCP | 37.4 (35.6-39.2)  32.0 (29.5-34.5)  45.0 (42.8-47.2)  43.0 (37.5-48.5) | 51.0 (49.3-52.7)  49.4 (47.7-51.1)  51.0 (48.8-53.2)  55.7 (52.4-59.0) | 13.6  17.4  6  12.7 | < 0.001  < 0.001  = 0.001  < 0.001 |
|  | | | | |
| Median age at diagnosis of diabetes mellitus (years (95% CI))  All patients  ICP  ACP  SCP | 51.1 (47.6-54.6)  51.0 (47.1-54.9)  56.5 (NA*)  NA | 67.0 (63.0-71.0)  67.0 (61.8-72.1)  64.0 (57.4-70.6)  NA | 15.9  16  7.5  NA | < 0.001  < 0.001  = 0.10  = 0.012 |
| Median age at diagnosis of steatorrhea (years (95% CI))  All patients  ICP  ACP  SCP | 61.0 (57.9-64.2)  61.0 (55.8-66.3)  71.9 (NA)  NA | NA  NA  65.8 (60.2-71.4)  NA | NA  NA  -6.1  NA | < 0.001  < 0.001  = 0.085  = 0.002 |

*NA, not applicable.
